# Electrowetting Using a Microfluidic Kelvin Water Dropper

**DOI:** 10.3390/mi9030092

**Published:** 2018-02-25

**Authors:** Elias Yazdanshenas, Qiang Tang, Xiaoyu Zhang

**Affiliations:** 1 Department of Mechanical & Aerospace Engineering, Old Dominion University, Norfolk, VA 23529, USA; eyazd001@odu.edu (E.Y.); tangqiang102@126.com (Q.T.); 2 State Key Lab of Mechanics and Control of Mechanical Structures, Nanjing University of Aeronautics & Astronautics, Nanjing, Jiangsu 210016, China

**Keywords:** microfluidic Kelvin water dropper, high voltage, COMSOL, electrowetting

## Abstract

The Kelvin water dropper is an electrostatic generator that can generate high voltage electricity through water dripping. A conventional Kelvin water dropper converts the gravitational potential energy of water into electricity. Due to its low current output, Kelvin water droppers can only be used in limited cases that demand high voltage. In the present study, microfluidic Kelvin water droppers (MKWDs) were built in house to demonstrate a low-cost but accurately controlled miniature device for high voltage generation. The performance of the MKWDs was characterized using different channel diameters and flow rates. The best performed MKWD was then used to conduct experiments of the electrowetting of liquid on dielectric surfaces. Electrowetting is a process that has been widely used in manipulating the wetting properties of a surface using an external electric field. Usually electrowetting requires an expensive DC power supply that outputs high voltage. However, in this research, it was demonstrated that electrowetting can be conducted by simply using an MKWD. Additionally, an analytic model was developed to simulate the electrowetting process. Finally, the model’s ability to well predict the liquid deformation during electrowetting using MKWDs was validated.

## 1. Introduction

The Kelvin water dropper was invented by William Thompson (aka. Lord Kelvin) in 1876 [1]. The Kelvin water dropper is an electrostatic generator that produces high voltage direct current (DC) by water dripping [1,2,3,4], in which potential energy is converted into electrical energy [5]. A typical Kelvin water dropper produces electrostatic charges by separating ions in water using two positive feedback loops. The output is usually controlled by the water flow rate and the placement of electrode components. The maximum output is limited by the ambient humidity and the quality of electrical insulation, both of which can lead to discharge. Since its invention, Kelvin water droppers have not drawn too much attention due to their low power output. Research on Kelvin water droppers is limited and they has been mainly used for classroom demonstrations [6,7,8,9]. In recent years, Planinšič and Prosen improved the voltage output of a Kelvin water dropper by placing the metal tube on the axis of the induction rings to transfer more charges from the Kelvin water dropper [10]. Özdemir et al. demonstrated that a Kelvin water dropper driven by pressurized water jets could also generate a high voltage up to 4 kV [11]. Huang investigated several parameters that affect the voltage output of a Kelvin water dropper. He reported that water flow rate, placement of the induction rings, and the size of the inductors significantly influence the voltage [12]. Generally, the voltage output of a conventional Kelvin water dropper is affected by many factors, including flow rate, the placement of the two inductors and two collectors, electrical insulation, and humidity. 

Although Kelvin water droppers provide a cheap way to produce electricity, they has very limited applications, due to their extremely low current output. Similar to other electrostatic generators invented in history, the Kelvin water dropper can only be used where high voltage but tiny or no current is needed. It was not until recently that the Kelvin water dropper has received interest again for its potential applications in microfluidic devices. In the last decade, with the extensive advances in microfluidic devices, researchers started implementing Kelvin water droppers in microfluidic devices for energy conversion and microfluidic manipulation [4,13,14,15]. Marín et al. demonstrated the use of a microfluidic Kelvin water dropper (MKWD) that is a lab-on-a-chip device to produce charged micro droplets at a high frequency using pneumatic pressure-driven flows. They improved the energy conversion efficiency by scaling down the Kelvin water dropper [13]. Xie et al. presented another pressure-driven MKWD to harvest direct current (DC) using diodes. Stable DC output was achieved, though the current was still at low magnitudes (i.e., in the range of nA). An equivalent circuit that includes two positive feedback loops was also constructed to simulate their experimental results [14]. Nevertheless, the reported devices created a new paradigm for the applications of Kelvin water droppers in microfluidics. 

To further explore the applications of Kelvin water droppers at a small scale, researchers have investigated the control parameters that can improve the performance of MKWDs [4,11]. An important application using the ultra-high voltage produced by MKWDs is electrowetting [16]. Electrowetting has been widely used in lab-on-a-chip devices for biomedical analysis and diagnostics [16], electronic display (e-paper) [17], tunable filter fibers [18], optical application [19], and microfluidic manipulation [20,21,22]. Electrowetting is a technique used to manipulate the wetting properties of a substrate under an external electric field [23]. The shape of a droplet on a surface can be controlled by manipulating the electric field strength across, resulting in various extents of surface wetting. Electrowetting generally requires high voltage yet minimum current, and the MKWD meets these demands perfectly while providing a low-cost solution.

In this paper, an MKWD is fabricated in house to output high voltage for the electrowetting of deionized (DI) water droplets on dielectric surfaces. The characteristics of the electrowetting system were evaluated experimentally and analytically, in order to demonstrate its feasibility. The control parameters of the MKWD, including flow rate, tube diameter, and placement of the inductors and collectors, were optimized for a more stable control of the electrowetting process. An analytic model was constructed to simulate the experimental results using COMSOL (COMSOL Inc., Stockholm, Sweden), a finite element analysis platform that can handle computational fluid dynamics (CFD). Hereby, a novel and low-cost method for electrowetting was demonstrated. To our best knowledge, electrowetting using MKWDs has rarely been addressed in the literature. Therefore, it requires intensive investigations on a theoretical framework, performance characterization, and systematical optimization. 

## 2. Methods

### 2.1. Experimental Method

**Figure 1:**
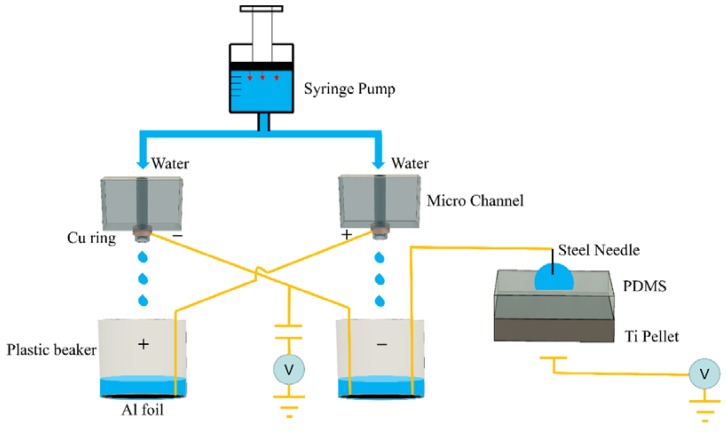
Schematic diagram of the electrowetting system using the microfluidic Kelvin water dropper (MKWD).

The schematic diagram of the electrowetting system using an MKWD is shown in Figure 1. The MKWD mainly consists of a syringe pump, two microfluidic channels, two inductors, and two collectors. DI water is injected by a syringe pump (Harvard Apparatus PHD 2000, Harvard Apparatus, Holliston, MA, USA) into two microfluidic channels made of polydimethylsiloxane (PDMS). The syringe pump was used to ensure stable flow control in the experiments. The water jets break into droplets after passing through the induction rings, which are made of copper and are well insulated by liquid electrical tape. The water droplets with electrostatic charges are collected by the aluminum foils placed at the bottom of the beakers. The inductors and collectors are cross-linked so that a positive feedback system is formed to generate a high potential difference between electrodes. Initially, there is a slight potential difference between two collectors by nature. Once water starts flowing, the inductors (either slightly positive or negative) attract the ions with the opposite charge, whereas they expel those with the same charge. Therefore, the more water droplets that are collected, the higher potentials the collectors can reach. This will result in the higher potentials of the inductors, which in turn further enhance the separation of the charges in water. Ultimately, the output potentials will reach a dynamically steady state where the leakage current counters the generated charges.

The electrowetting setup consists of a working electrode, a dielectric substrate, and a counter electrode. The working electrode made of a steel needle (0.31 mm in diameter) was inserted into the droplet. Ideally, the needle should be placed exactly at the center of the water droplet to prevent any movement of the water droplet due to charge imbalance. An electric field obtained from the output of the MKWD was applied through the needle. The counter electrode was made of a 0.9-mm thick titanium (Ti) pellet. The Ti pellet was coated with a thin layer of PDMS, which serves as a dielectric substrate. Ti pellet was selected due to its physiochemical stability under high voltage [24] and good color contrast to the droplet for better video quality. PDMS is an excellent dielectric material that can provide a hydrophobic layer suitable for electrowetting. All surface voltage (i.e., electrostatic voltage) measurements were performed via an electrostatic voltmeter (Model SVMII, AlphaLab, Salt Lake City, UT, USA). 

The microchannels of MKWDs were fabricated through a molding process, in which two long steel needles were embedded into a PDMS substrate. The PDMS substrates were cured for 8 h on a hot plate at 80 °C. Thereafter, the needles were removed to create the microchannels. Needles with different diameters (254 µm and 508 µm) were used in order to investigate the sizing effect on the output of MKWDs. The surface of the Ti pellet was cleaned with ethanol in an ultrasonic bath for 15 min. To prepare PDMS, the base elastomer and curing agent were well mixed at a 10:1 ratio, followed by resting in air for 1.5 h for eliminating bubbles. To fabricate the electrowetting setup, a Ti pellet was coated by PDMS to form a hydrophobic dielectric layer using a spin-coater (Model VTC-100, MTI Corp., Richmond, CA, USA) at the speed of 500 revolutions per minute (rpm) for 4 s. Then, the coated Ti pellet was air dried for 8 h on a hot plate at 80 °C.

Performance characterization of the fabricated MKWDs was carried out, in order to identify the optimized operating parameters of MKWDs for electrowetting. High voltage generation, fast response time, and stable output were used as the criteria for selecting the appropriate operating parameters. The output of two MKWDs with different tube inner diameters (IDs) (254 µm and 508 µm) were investigated under different flow rates. 

Table 1 lists the operating conditions of the MKWDs investigated. The experiment sets A1 and A2 represent the operating parameters associated with two MKWDs with different IDs. After the performance characterization, the best-performing MKWD with the optimized flow rate was used to carry out experiments on electrowetting (experiment B), with the operating parameters also listed in Table 1. In all of the experiments, the distance between the center of the right and left inductors, and the distance between the end of the microfluidics tubes and the center of the two collectors are 60 cm, 40 cm, and 60 cm, respectively. 

### 2.2. Analytical Method

**Figure 2:**
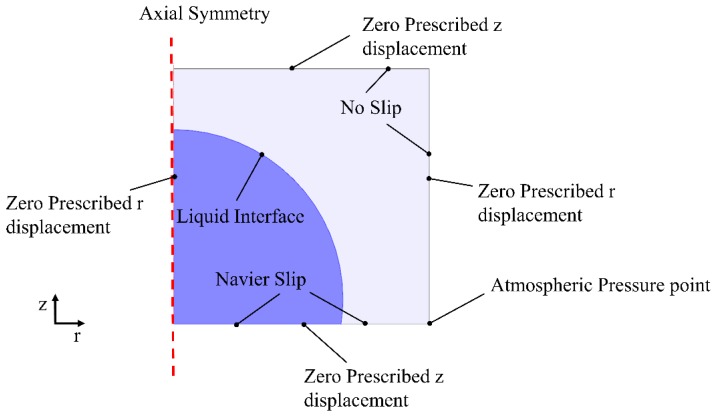
Simulation model of the droplet deformation on a polydimethylsiloxane (PDMS) substrate during electrowetting.

In the present work, an analytical model was built to simulate the time-dependent deformation of a water droplet during electrowetting using MKWDs. A COMSOL microfluidics module was used to carry out the simulation. The simulation results were compared to the experimental observation side by side, so as to validate the model. Figure 2 illustrates the model adopted in the simulation, in which an axisymmetric configuration was used, assuming that the shape of the droplet is a perfect hemisphere [25]. The initial diameter of the water droplet in the model was set at 2.44 mm, in alignment with the actual size used in the experiment for comparison. The droplet was fixed on a dielectric PDMS substrate with a thickness of around 30 µm, to ensure that no dielectric breakdown would occur in the present experiments [26]. Zero prescribed z displacement was applied as the boundary conditions to the top and bottom edges. Also, zero prescribed r displacement was applied as the boundary conditions to the left and right edges. Navier slip was applied to the bottom boundary due to the hydrophobic property of the PDMS layer, and the associated frictional force can be expressed as:

(1) $$F_{\mathrm{fr}}=-\mu \frac{u}{\beta }$$

where *µ*, **u**, and *β* denote the viscosity of the fluid, fluid velocity, and slip length, repectively. In addition, non-slip was applied to both the right and the top boundaries, within which air is confined. Finally, a liquid interface was set as the boundary between the water droplet and air. The contact angle is defined as the angle formed between the tangent of inside the water droplet and the bottom substrate [27,28].

To solve this problem analytically, the two-phase compressible flow Navier-Stokes equation was adapted [29,30] . Hence, Laminar Tow-Phase Flow Moving Mesh in COMSOL was selected [31]:

(2) $$\rho \frac{\partial u}{\partial t}+\rho \left(u\cdot \nabla \right)u=\nabla \left[-pI+\mu \left(\nabla u+{\left(\nabla u\right)}^{T}\right)-\frac{3}{2}\mu \left(\nabla \cdot u\right)I\right]+F_{s}+\;F_{b}$$

(3) $$\rho \frac{\partial u}{\partial t}+\nabla \left(\rho u\right)=0$$

where **u**, ρ, *μ*, *p*, $F_{s}$, and $F_{b}$ denote the velocity field, mass density, dynamic viscosity, pressure, surface force per unit volume, and body force per unit volume. $F_{s}$ can be expressed as [28]:

(4) $$F_{s}=\nabla \cdot \sigma \left(I-{\left(nn\right)}^{T}\right)\delta$$

where **I**, *σ*, **n**, and δ represent the identity matrix, co-efficient of surface tension, interface normal direction, and direct delta function, respectively. The direct delta function can be defined as:

(5) $$\delta =\{\begin{array}{c} \frac{\left(1+\cos\left(2\pi \emptyset \right)\right)}{3h}\;if\;\left|\emptyset \right|\leq 1.5h\\ 0\;\;\;otherwise \end{array}$$

where *h* and ∅ are the grid spacing and level set function. The level set function is a function of the lowest distance from the center of the droplet to the interface and time, as determined below:

(6) $$\emptyset \left(x,t\right)=\{\begin{array}{c} >0\;\;\;outside\;of\;the\;interface\\ =0\;\;\;\;at\;the\;interface\\ <0\;\;\;inside\;of\;the\;interface \end{array}$$

$F_{b}$ in the present scenario is only attributed to electric force, whereas gravity is neglected. Moon et al. reported that the surface tension of the interface in electrowetting was affected by both electrical and chemical segments [32]. Those segments were created by the potential difference between the water droplet and the substrate, which can be described using Lippmann’s equation [32]. The electric segment was introduced as a capacitor, whereas the chemical segment was defined as the natural surface tension of the droplet on the interface. Additionally, Lipmann’s equation was derived from the Gibbsian interfacial thermodynamic analysis of the interface. Hence, this equation was implemented in the present simulation due to the similarities in our experimental setup. As mentioned before, the electric field on the solid-liquid interface affects the amount of charged counter ions in the water droplet, and their relationship can be explained in Equation (7) [33]:

(7) $$\gamma_{\mathrm{SL}}=-\rho_{\mathrm{sl}}\;dV=-\int_{v_{p}}^{v}\rho_{\mathrm{sl}}\;dV$$

(8) $$\rho_{\mathrm{sl}}=\int_{v_{p}}^{v}c\;dV$$

where $\gamma_{\mathrm{SL}},$ $\rho_{\mathrm{sl}}$, *V*, $v_{p}$, and c are the surface tension of solid-liquid interfaces, the surface charge density of counter-ions, the applied voltage, the voltage of the trapped charge, and the capacitance across the interface, respectively. By substituting Equation (8) into Equation (7), $\gamma_{\mathrm{SL}}$ can be expressed as [32]:

(9) $$\gamma_{\mathrm{SL}}=\iint_{v_{p}}^{v}cdVdV=\gamma_{0}-\frac{1}{2}cV^{2}$$

where $\gamma_{0}$ is the surface tension of the solid-liquid interface at the potential zero charge. In Lippman’s equation (as shown below), the contact angle is affected by the surface tensions of interfaces, including liquid-gas ($\gamma_{\mathrm{LG}}$), solid-gas ($\gamma_{\mathrm{SG}}$), and solid-liquid ($\gamma_{\mathrm{SL}}$).

(10) $$\gamma_{\mathrm{LG}}\;\cos\theta =\gamma_{\mathrm{SG}}-\gamma_{\mathrm{SL}}$$

where θ is the contact angle. Then, Lipmann’s equation can be written as [32]:

(11) $$\cos\theta =\cos\theta_{0}+\frac{1}{\gamma_{\mathrm{LG}}}\frac{1}{2}cV^{2}$$

where $\theta_{0}$ is the initial contact angle of the liquid-solid interface prior to applying any electric field. The above equation indicates that the contact angle depends on several parameters, including the orignial state, the surface tension of liquid-gas ($\gamma_{\mathrm{LG}}$) which refers to water and air in the present case, applied voltage (*V*) on the water droplet, and the interface capacitance (*c*) per unit area, which is expressed below [32]:

(12) $$c=\frac{\varepsilon_{0}\varepsilon_{r}}{d}$$

where *ε*_r_, *ε*_0_, and *d* are the relative permittivity, vacuum permittivity, and thickness of the dielectric, respectively. The expression of the contact angle can be derived from Equations (7) and (8), as shown below:

(13) $$\theta =\arccos(\cos\theta_{0}+\frac{1}{\gamma_{\mathrm{LG}}}\frac{\varepsilon_{0}\varepsilon_{r}}{2d}V^{2})$$

In the simulation, $\theta_{0}$ was set at 90° in alignment with the initial angle observed in the experiments. Other parameters, including $\gamma_{\mathrm{LG}}$, *ε*_r_, and *d*, were set at 0.072 N/m [34],

2.56 [35], and 30 µm, respectively. The time-dependent voltage used in the simulation was obtained from the curve fitting of the voltage curve logged during the experiments.

## 3. Results and Discussion

### 3.1. Performance Evaluation of the Microfluidic Kelvin Water Droppers (MKWDs)

**Figure 3:**
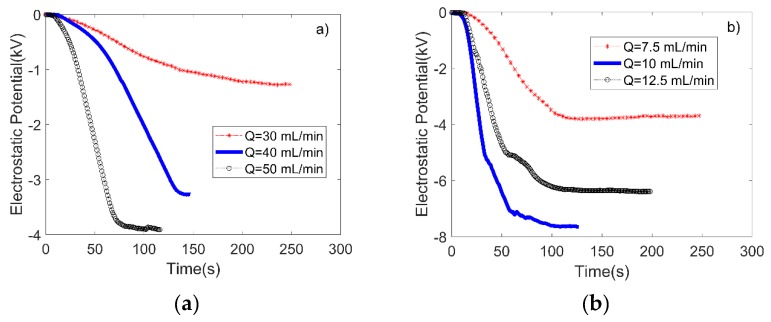
Output voltages of the negative electrodes of two MKWDs over a period up to 250 s. (**a**) and (**b**) represent the results from the MKWDs with microchannel IDs of 508 µm and 254 µm, respectively. The calculated average flow speeds were 2.517 (red), 3.356 (blue), and 4.195 (black) m/s for both MKWDs.

In the experiments A1 and A2, the performance of the MKWDs were evaluated to identify the optimized operating conditions with the highest voltage output for use in electrowetting. Figure 3 shows the outputs of the MKWDs with two different microchannel diameters using various flow rates. The duration of each experiment depends on the volumetric capacity of the syringe pump and the flow rate. Figure 3 only shows the data collected from the negative electrodes of MKWDs. Two phenomena were observed from the experiments. First, for both MKWDs the output voltages increased as the flow rate increased. The charge collectors in the MKWD can be considered as a capacitor. Therefore, in the experiments, higher flow rate led to a larger charging current and faster charging rate. As a result, a higher voltage gain and shorter charging time were achieved. Second, the output voltages plateaued at certain voltage levels. As the voltage increased, the charge leakage became more and more significant. The output voltage can only reach a point where the leaking current counters the charging current. This means that the net gain of charges vanishes and therefore the output voltage remains constant. The highest output voltages from both MKWDs were obtained with the highest flow rate at 12.5 mL/min and 50 mL/min, respectively. The maximum voltage recorded was around −7700 V, which was obtained from the MKWD with the smaller ID (254 μm) at 12.5 mL/min. It was an unexpected and interesting phenomenon that the microchannel dimension was found to have a huge impact on voltage output. A possible explanation for this is that, for electrostatic sprays, a larger flow rate results in a much larger droplet size, and thus may cause a lower amount of charge carriers [36]. However, the sizing effect on the output of MWKDs requires further investigation. Nevertheless, it was successfully demonstrated that MWKDs can provide ultra-high and stable voltage output for electrowetting.

It was found that the charge leakage becomes a dominant factor that limits the ultimate output voltage of the MKWD. Ambient humid air and insulating materials are the main causes of charge leakage. Water vapor in air has a tendency to remove charges from objects. Higher humidity means higher water content in air, which results in an increase of its electrical conductivity, making discharging easier [37]. People usually observe electrostatic sparks or shocks in winter, because the humidity reaches its lowest level in winter, which minimizes discharge. The insulating materials and their thickness also affect the voltage output. Although the Ohmic resistance of an insulator is high, the leaking current may become significant if the voltage increases to a significant level. In addition, if dielectric breakdown of the insulator occurs, it may cause a catastrophic failure of insulation and thus generate unsteady voltage output. Therefore, in order to obtain a high voltage and stable output, humidity control and appropriate electrical insulation are critical. Specifically, in our experiments, humidity was maintained using an electrical fan and monitored by a humidity meter. Thick layers of liquid electrical tape were applied to provide good electrical insulation.

### 3.2. Electrowetting Using the Microfluidic Kelvin Water Dropper (MKWD)

After performance evaluation, the MKWD with a smaller microfluidic channel (ID = 254 µm) was selected to supply voltage for conducting the electrowetting of DI water droplets on a PDMS substrate. Water droplets on a dielectric substrate will deform under an applied electric field. The deformed shape is attributed to several factors, including the strength of the electric field, the thickness of the dielectric substrate, the placement of electrodes, and the ambient environment that may cause discharge. In the present research, note that the voltage measurements were conducted beneath the counter electrode (Ti pellet) instead of the working electrode (steel needle), where accurately measuring the surface voltage was difficult.

**Figure 4:**
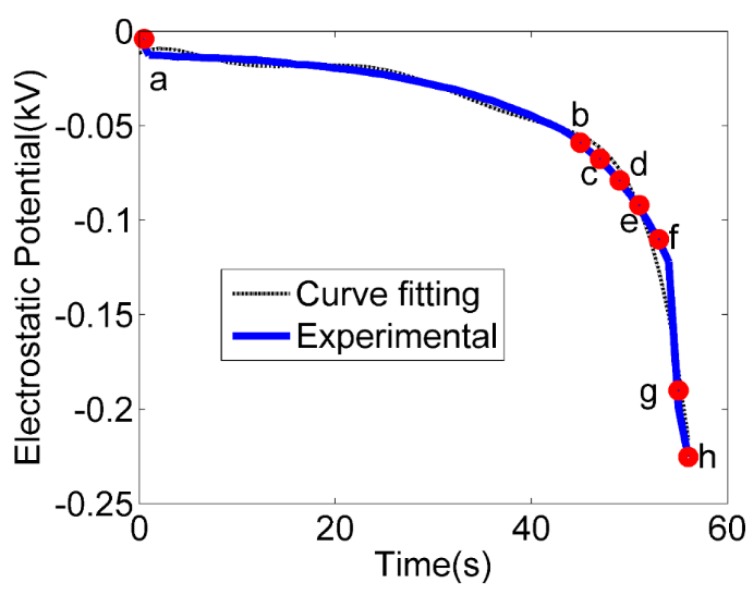
Voltage measurements (solid blue line) during electrowetting and a curve (dotted black line) that fits the experimental data.

Figure 4 shows the voltage data (blue line) recorded from the counter electrode during a typical electrowetting test. The operating conditions are shown in Experiment B in Table 1. The dotted line was obtained from the curve fitting of the experimental data to obtain a time-dependent function of voltage for simulation. Eight check points were selected to compare the experimental versus simulation results of the droplet deformation during the electrowetting test. 

**Figure 5:**
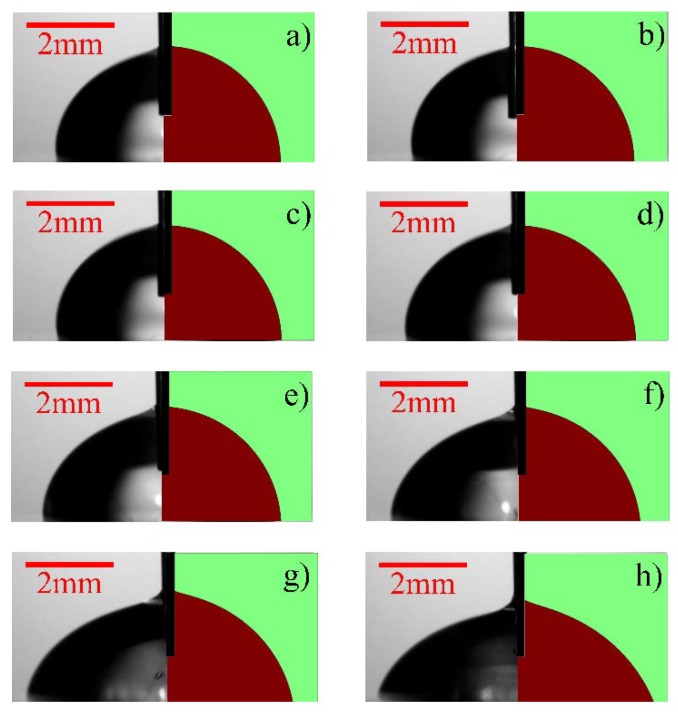
Time-lapse images of the experimental results (**left halves of the images**) versus the simulated water droplet deformation (**right halves of the images**) during the electrowetting test shown in Figure 4.

The left halves of the photos in Figure 5 show the time-lapse images of the electrowetting experiment, corresponding to the eight check points selected. The images were obtained from the frames of a video (Supplemental Video S1) recorded during the test by an optical microscope (AmScope OMAX A3560U, Amscope, Irvine, CA, USA). It was observed that the droplet remained almost unchanged until time passed point b, after which the deformation became more and more obvious. This was the reason why more check points were selected towards the end. Physically, electrowetting begins when the electric repulsion force starts to overcome the initial surface tension between the water and the PDMS substrate. The MWKD was used to charge the water droplet continuously through the needle, resulting in a hike of electric repulsion force. The net outcome was that the water droplet kept wetting more areas of the PDMS substrate. Another phenomenon observed in most experiments was that the electrowetting process only lasted up to 60 s, after which the droplet was ejected out like a bullet (shown in the supplemental video). This was because the cumulative charge imbalance overcame the overall attraction from the PDMS substrate. Even though the needle was carefully placed into the center of the droplet, ejection could hardly be prevented. In real applications, constraints such as a retention wall can be fabricated around the desired wetting area to retain the liquid. 

The right halves of the photos in Figure 5 show the CFD simulation results of those eight selected timepoints (shown in Figure 4). The animation of the whole simulation can be find in Supplemental Video S1. Because the applied voltage for electrowetting was obtained from the MWKD, a time-dependent voltage function was adapted in the simulation, unlike other research that used constant voltage values [25]. It can be seen that the simulation results closely match the experimental observations. The slight discrepancy between two images at point b was believed to be caused by the initial tension between the needle and water, which was not considered in the model. In general, the present model well predicted the behaviors of the droplet during electrowetting. The COMSOL simulation report was attached in Supplemental S2 for reference. 

**Figure 6:**
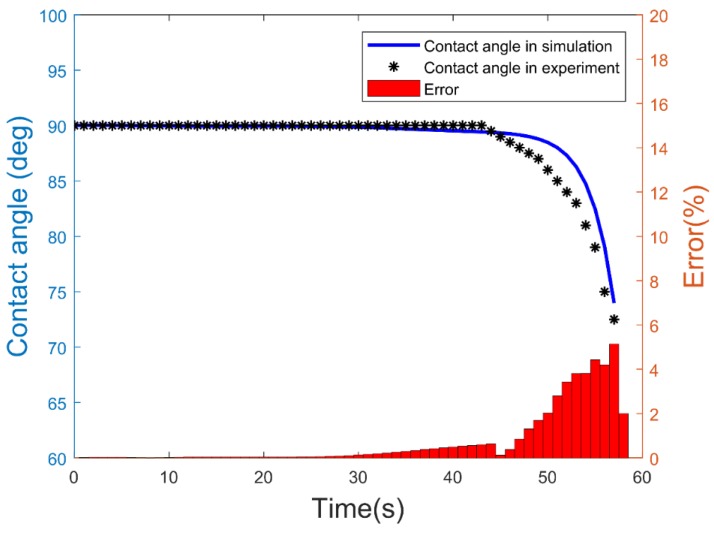
Change of the contact angle during electrowetting in the experiment versus the simulation, shown in Figure 4.

Figure 6 shows the change in the contact angle from the experiment and the simulation shown in Figure 4. It can be seen that the model constructed was able to simulate the experimental results with less than 6% error towards the end of electrowetting. The errors were calculated as follows:

(14) $$\mathrm{Error}=100\%\times \left|\frac{\theta_{\mathrm{Experiment}}-\theta_{\mathrm{Simulation}}}{\theta_{\mathrm{Experiment}}}\right|$$

The errors in the simulation were more likely caused by the discrepancies from the curve fitting of the experimental data points.

## 4. Conclusions

Microfluidic Kelvin water droppers were built in house to evaluate their feasibility for use in electrowetting. Ultra-high and stable voltage outputs were obtained from the MKWDs. The highest voltage recorded was 7700 V, which was obtained from the negative electrode of one MKWD that has 254 µm ID microchannels. The best performed MKWD was then used to conduct electrowetting tests. A combined analytical and experimental investigation was performed to characterize the droplet deformation during electrowetting. It was demonstrated that the MWKD is indeed a feasible and low-cost power supply that can be used in electrowetting. Furthermore, the experimental observations were well predicted by the numerical model presented in this paper. For future applications, electrowetting using MKWDs can be further optimized by environmental control (e.g., humidity, temperature), microfluidic flow management, and voltage output control.

## Figures and Tables

**Table 1:** Operating conditions of the microfluidic Kelvin water droppers (MKWDs).

Experimental Sets	Channel Inner Diameter (ID) (µm)	Water Flow Rate (mL/min)	Ambient Humidity (%)	Water Flow Speed (m/s)	Ambient Temperature (°C)
A1	508	30	48 ± 3	2.517	22
		40		3.356	
		50		4.195	
A2	254	7.5	48 ± 3	2.517	22
		10		3.356	
		12.5		4.195	
B	254	12.5	48 ± 3	4.195	22

## Supplementary Materials

The following are available online at http://www.mdpi.com/2072-666X/9/3/92/s1, Supplemental Video S1: Electrowetting using MKWD, Supplemental S2: COMSOL Simulation Report.

## Nomenclature

**u**	velocity field
ρ	mass density
*μ*	dynamic viscosity
*p*	pressure
$F_{s}$	surface force per unit volume
$F_{b}$	body force per unit volume
**I**	identity matrix
*σ*	coefficient of surface tension
**n**	interface normal direction
δ	direct delta function
*h*	grid spacing
∅	level set function
*E*	electric field
*V*	electrostatic voltage
$\theta_{\mathrm{Experiment}}$	contact angle in experiment
$\theta_{\mathrm{Simulation}}$	contact angle in simulation
