# Supplementary material for: Electrowetting Using a Microfluidic Kelvin Water Dropper
Source: Micromachines (Basel). 2018 Feb 25;9(3):92. doi: 10.3390/mi9030092 (PMC6187503; doi:10.3390/mi9030092)
Supplement: Supplementary file 1 [file micromachines-09-00092-s001.zip › micromachines-258371 supplementary/COMSOL Simulation Report.pdf]

Untitled

Report date	Feb 13, 2018 4:24:14 PM

Contents

1. Global Definitions......3
1.1. Parameters 1......3
2. Component 1......4
2.1. Definitions......4
2.2. Geometry 1......5
2.3. Materials......7
2.4. Laminar Two-Phase Flow, Moving Mesh......14
2.5. Mesh 1......43
3. Study......45
3.1. Phase Initialization......45
3.2. Time Dependent......45
3.3. Solver Configurations......46
4. Results......53
4.1. Data Sets......53
4.2. Tables......57
4.3. Plot Groups......58

1 Global Definitions

Date	Mar 1, 2017 1:31:30 PM

Global settings

Name	Final electrowetting.mph
Path	H:\Final electrowetting.mph
COMSOL version	COMSOL 5.1 (Build: 145)
Unit system	SI

Used products

COMSOL Multiphysics
Microfluidics Module

1.1 Parameters 1

Parameters

Name	Expression	Value	Description
theta0	90[deg]	1.5708 rad	Zero voltage contact angle
gamma	0.072[N/m]	0.072 N/m	Surface tension
epsr	2.65	2.65	Relative dielectric constant
d_f	0.03[mm]	3E−5 m	Dielectric thickness

2 Component 1

Date	Jan 25, 2017 12:51:21 PM

Component settings

Unit system	SI
Geometry shape order	automatic

2.1 Definitions

2.1.1 Variables

Variables 1

Selection

Geometric entity level	Entire model

Name	Expression	Unit	Description
theta	acos(cos(theta0) + V^2*epsr*epsilon0_const/(2*gamma*d_f))		Contact angle
V	-3.909655708607368E-7[V/s]*t^6 + 5.660499334244992E-5[V/s]*t^5 - 0.003054451422720[V/s]*t^4 + 0.074539710922610[V/s]*t^3 - 0.801776710957060[V/s]*t^2 + 2.533455101584655[V/s]*t - 11.530789606238486		

2.1.2 Coordinate Systems

Boundary System 1

Coordinate system type	Boundary system
Tag	sys1

Coordinate names

First (t1)	Second (to)	Third (n)
t1	to	n

Settings

Description	Value
Create first tangent direction from	Global Cartesian (spatial)

2.2 Geometry 1

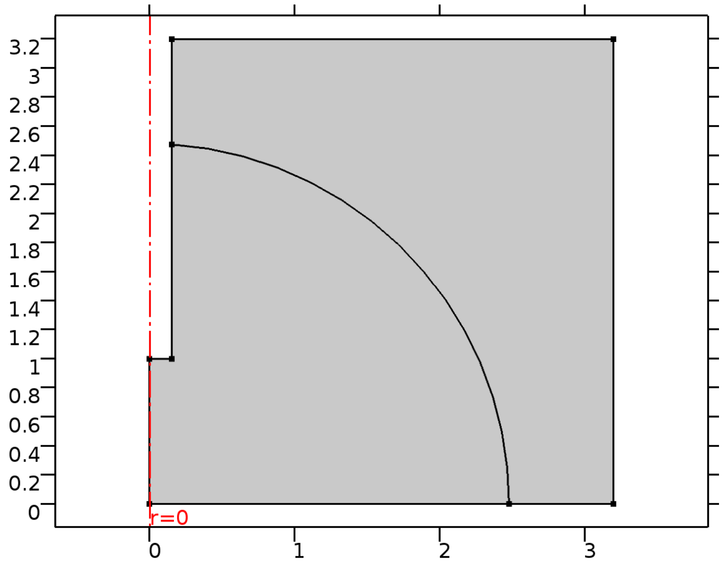

Geometry 1

Units

Length unit	mm
Angular unit	deg

Geometry statistics

Description	Value
Space dimension	2
Number of domains	2
Number of boundaries	9
Number of vertices	8

2.2.1 Rectangle 1 (r1)

Position

Description	Value
Position	{0, 0}
Layers	

Size

Description	Value
Width	3.2
Height	3.2

2.2.2 Circle 1 (c1)

Position

Description	Value
Position	{0, 0}
Layers	

Size and shape

Description	Value
Radius	2.48
Sector angle	90

2.2.3 Rectangle 2 (r2)

Position

Description	Value
Position	{0, 1}
Layers	

Size

Description	Value
Width	0.155
Height	2.7

2.3 Materials

2.3.1 Air

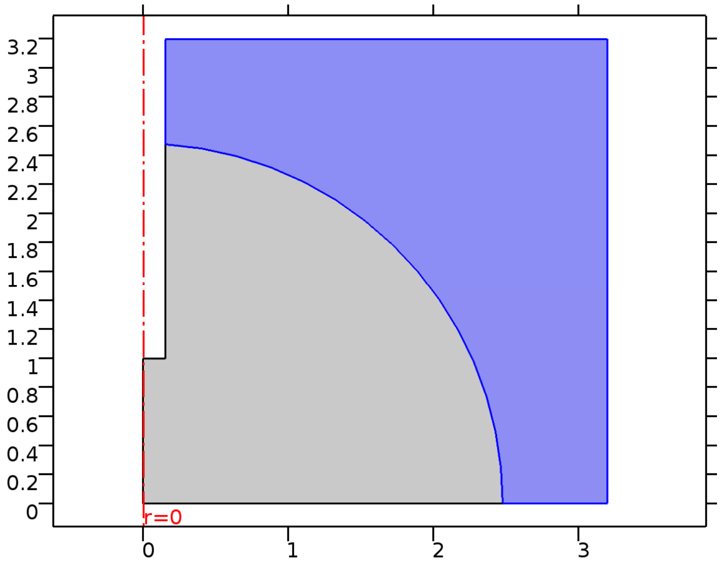

Air

Selection

Geometric entity level	Domain
Selection	Domain 2

Material parameters

Name	Value	Unit
Dynamic viscosity	eta(T[1/K])[Pa*s]	Pa*s
Density	rho(pA[1/Pa],T[1/K])[kg/m^3]	kg/m^3

Basic Settings

Description	Value
Relative permeability	{{1, 0, 0}, {0, 1, 0}, {0, 0, 1}}
Relative permittivity	{{1, 0, 0}, {0, 1, 0}, {0, 0, 1}}
Dynamic viscosity	eta(T[1/K])[Pa*s]
Ratio of specific heats	1.4
Electrical conductivity	{{0[S/m], 0, 0}, {0, 0[S/m], 0}, {0, 0, 0[S/m]}}
Heat capacity at constant pressure	Cp(T[1/K])[J/(kg*K)]
Density	rho(pA[1/Pa], T[1/K])[kg/m^3]
Thermal conductivity	{{k(T[1/K])[W/(m*K)], 0, 0}, {0, k(T[1/K])[W/(m*K)], 0}, {0, 0, k(T[1/K])[W/(m*K)]}}

Description	Value
Speed of sound	cs(T[1/K])[m/s]

Functions

Function name	Type
eta	Piecewise
Cp	Piecewise
rho	Analytic
k	Piecewise
cs	Analytic

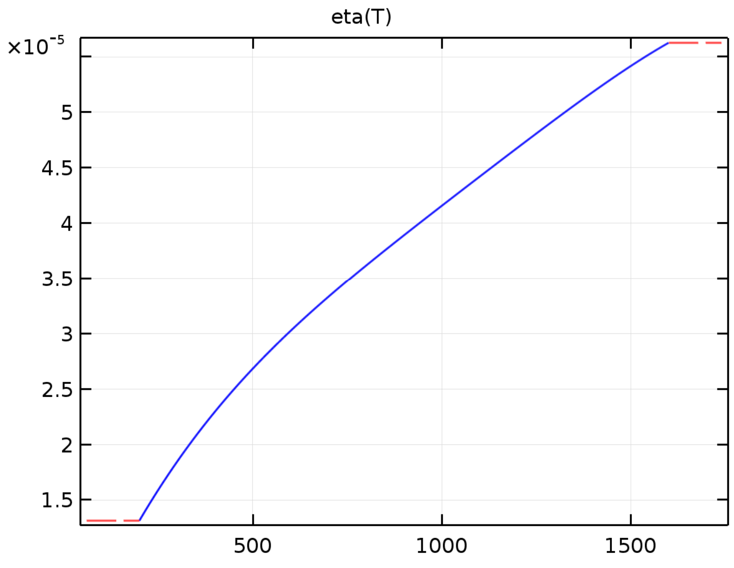

eta

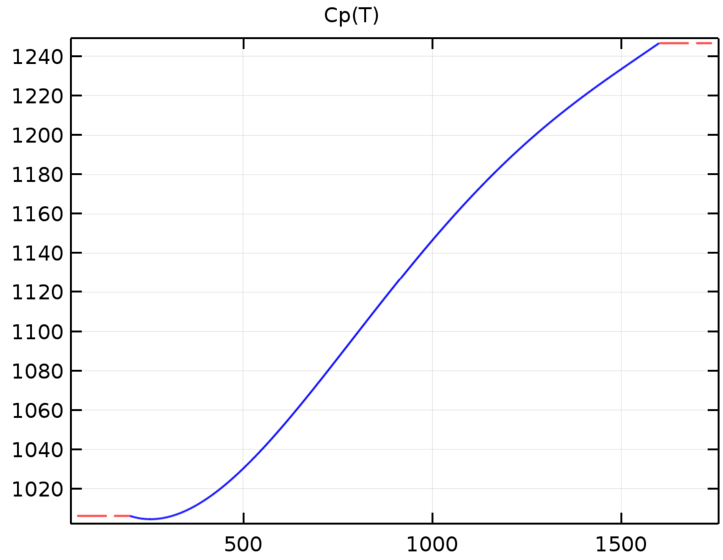
Cp(T)
1240
1220
1200
1180
1160
1140
1120
1100
1080
1060
1040
1020
500
1000
1500

Cp

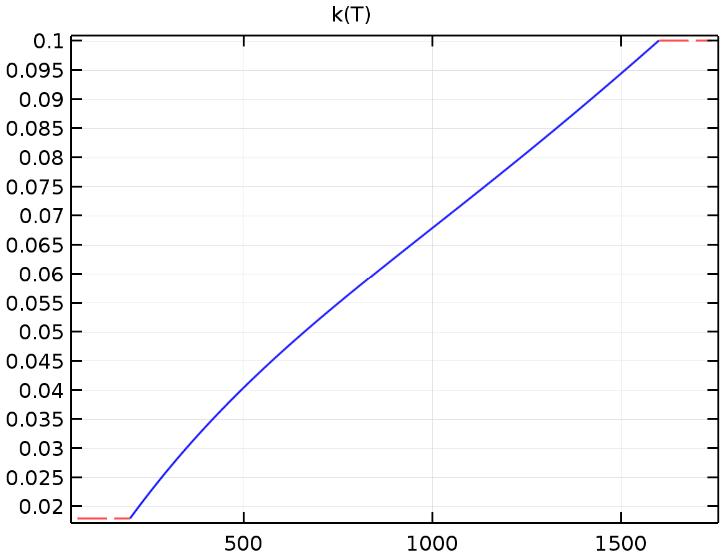
k(T)
0.1
0.095
0.09
0.085
0.08
0.075
0.07
0.065
0.06
0.055
0.05
0.045
0.04
0.035
0.03
0.025
0.02
500
1000
1500

k

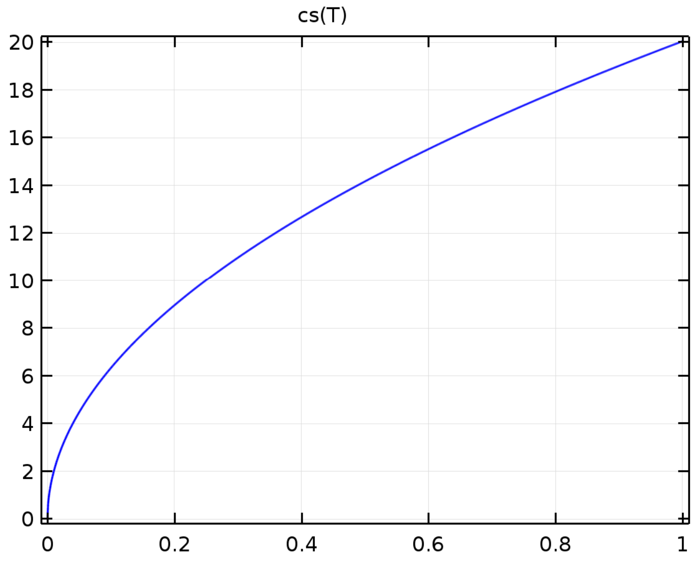

cs

Refractive index Settings

Description	Value
Refractive index	{{1, 0, 0}, {0, 1, 0}, {0, 0, 1}}
Refractive index, imaginary part	{{0, 0, 0}, {0, 0, 0}, {0, 0, 0}}

2.3.2 Water, liquid

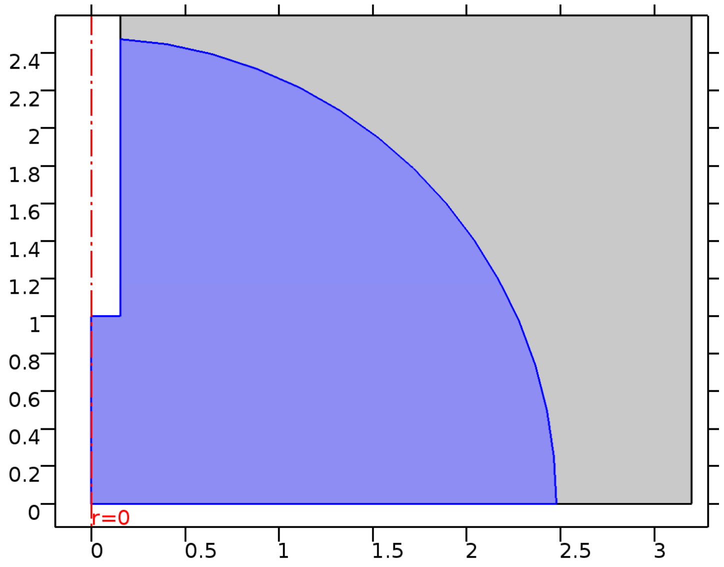

Water, liquid

Selection

Geometric entity level	Domain

Selection	Domain 1

Material parameters

Name	Value	Unit
Dynamic viscosity	eta(T[1/K])[Pa*s]	Pa*s
Density	rho(T[1/K])[kg/m^3]	kg/m^3

Basic Settings

Description	Value
Dynamic viscosity	eta(T[1/K])[Pa*s]
Ratio of specific heats	1.0
Electrical conductivity	{{5.5e-6[S/m], 0, 0}, {0, 5.5e-6[S/m], 0}, {0, 0, 5.5e-6[S/m]}}
Heat capacity at constant pressure	Cp(T[1/K])[J/(kg*K)]
Density	rho(T[1/K])[kg/m^3]
Thermal conductivity	{{k(T[1/K])[W/(m*K)], 0, 0}, {0, k(T[1/K])[W/(m*K)], 0}, {0, 0, k(T[1/K])[W/(m*K)]}}
Speed of sound	cs(T[1/K])[m/s]
Relative permittivity	{{80, 0, 0}, {0, 80, 0}, {0, 0, 80}}

Functions

Function name	Type
eta	Piecewise
Cp	Piecewise
rho	Piecewise
k	Piecewise
cs	Interpolation

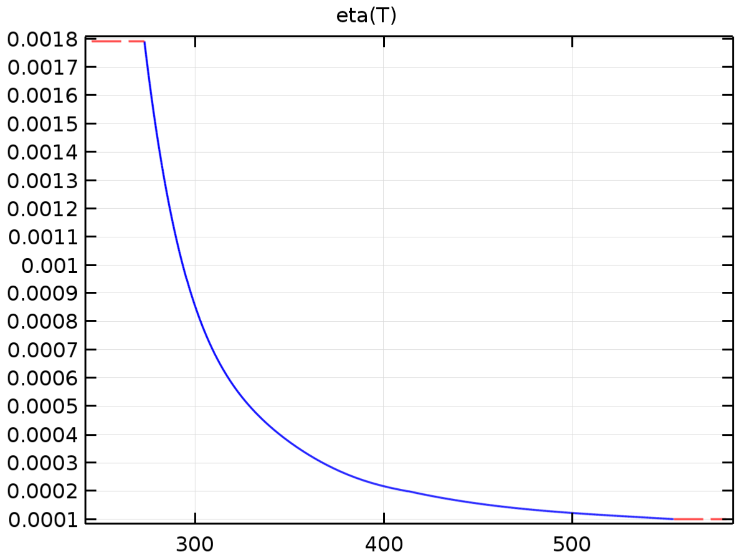
eta(T)
0.0018
0.0017
0.0016
0.0015
0.0014
0.0013
0.0012
0.0011
0.001
0.0009
0.0008
0.0007
0.0006
0.0005
0.0004
0.0003
0.0002
0.0001
300
400
500

eta

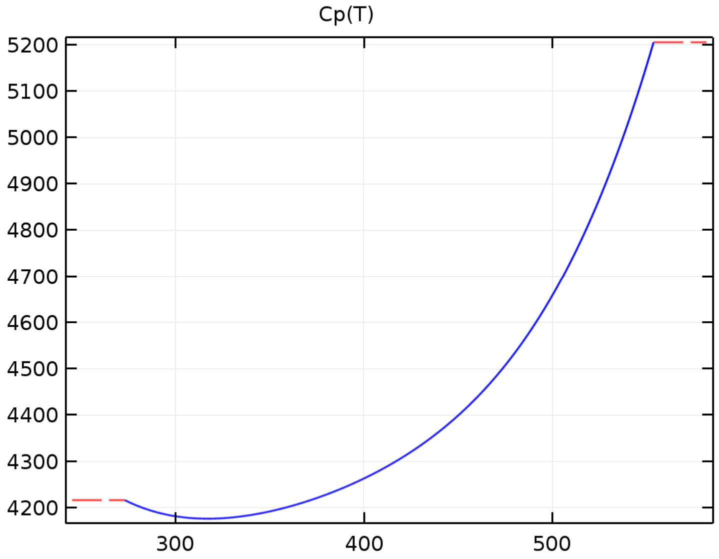
Cp(T)
5200
5100
5000
4900
4800
4700
4600
4500
4400
4300
4200
300
400
500

Cp

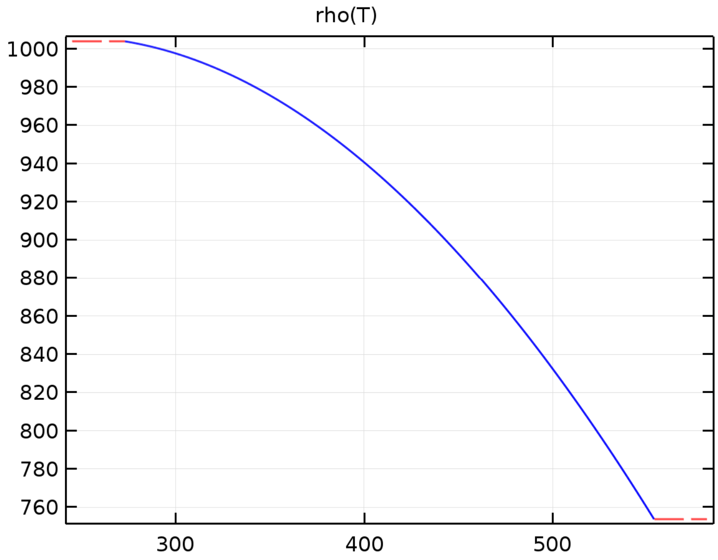
rho(T)
1000
980
960
940
920
900
880
860
840
820
800
780
760
300
400
500

rho

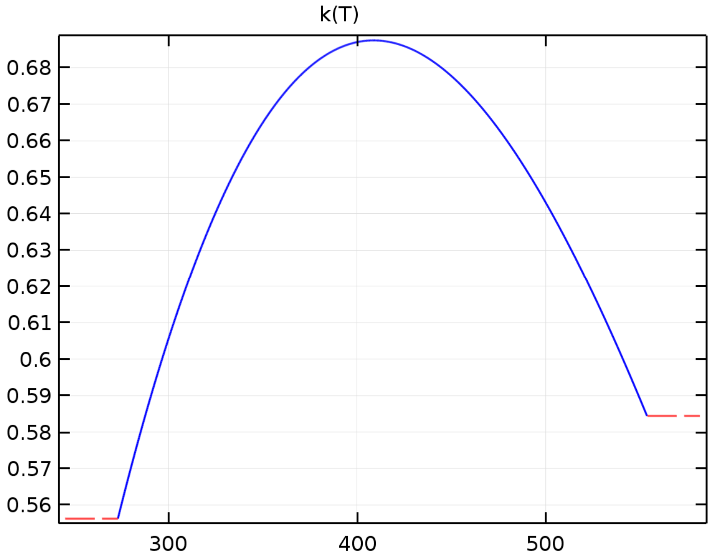
k(T)
0.68
0.67
0.66
0.65
0.64
0.63
0.62
0.61
0.6
0.59
0.58
0.57
0.56
300
400
500

k

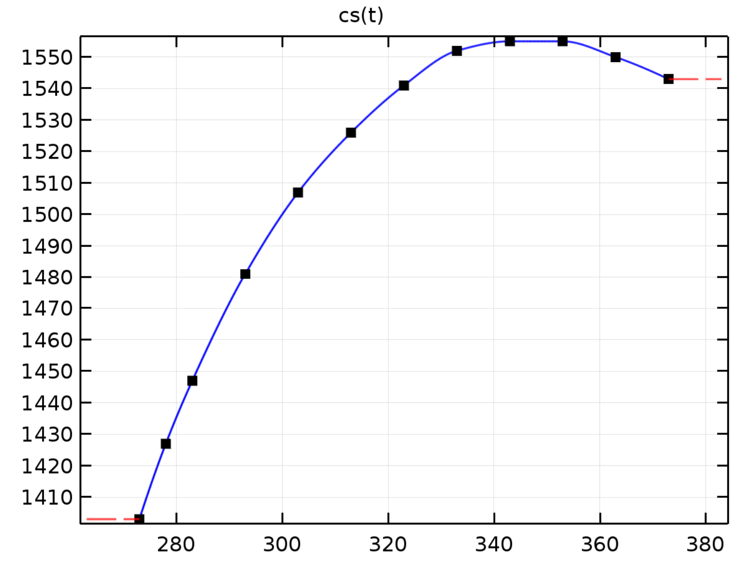

cs

2.4 Laminar Two-Phase Flow, Moving Mesh

Used products

COMSOL Multiphysics
Microfluidics Module

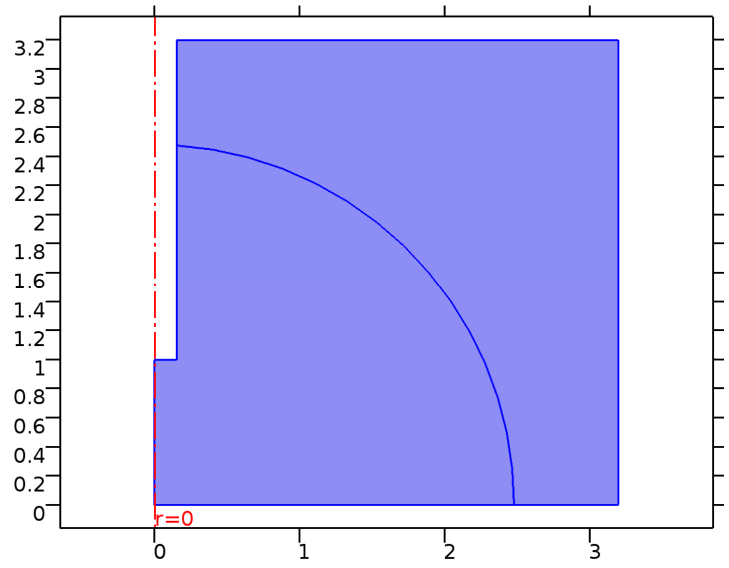

Laminar Two-Phase Flow, Moving Mesh

Selection

Geometric entity level	Domain
Selection	Domains 1–2

Equations

$$\rho\frac{\partial \mathbf{u}}{\partial t}+\rho(\mathbf{u}\cdot\nabla)\mathbf{u}=$$
$$\nabla\cdot\left[-p\mathbf{I}+\mu\left(\nabla\mathbf{u}+(\nabla\mathbf{u})^T\right)-\frac{2}{3}\mu(\nabla\cdot\mathbf{u})\mathbf{I}\right]+\mathbf{F}$$
$$\frac{\partial\rho}{\partial t}+\nabla\cdot(\rho\mathbf{u})=0$$

Settings

Description	Value
Discretization of fluids	P1 + P1
Value type when using splitting of complex variables	{Real, Real}
Material frame coordinates	{R, PHI, Z}
Geometry shape order	1

Variables

Name	Expression	Unit	Description	Selection
tpfmm.nr	nr		Normal vector, r component	Boundaries 1–9
tpfmm.nphi	root.nphi		Normal vector, phi component	Boundaries 1–9
tpfmm.nz	nz		Normal vector, z component	Boundaries 1–9
tpfmm.nrmesh	root.nrmesh		Normal vector (mesh), r component	Boundaries 1–9
tpfmm.nphimesh	root.nphimesh		Normal vector (mesh), phi component	Boundaries 1–9
tpfmm.nzmesh	root.nzmesh		Normal vector (mesh), z component	Boundaries 1–9
tpfmm.nR	nR		Normal vector, R component	Boundaries 1–9
tpfmm.nPHI	root.nPHI		Normal vector, PHI component	Boundaries 1–9

Name	Expression	Unit	Description	Selection
tpfmm.nZ	nZ		Normal vector, Z component	Boundaries 1–9
tpfmm.nRmesh	root.nRmesh		Normal vector (mesh), R component	Boundaries 1–9
tpfmm.nPHImesh	root.nPHImesh		Normal vector (mesh), PHI component	Boundaries 1–9
tpfmm.nZmesh	root.nZmesh		Normal vector (mesh), Z component	Boundaries 1–9
tpfmm.relVol	(d(r,root.xi1)*d(z,root.xi2)-d(r,root.xi2)*d(z,root.xi1))/dvol	1	Local relative element volume	Domains 1–2
tpfmm.relVolMin	tpfmm.minOp(tpfmm.relVol)	1	Minimum relative element volume	Global
tpfmm.relVolMax	tpfmm.maxOp(tpfmm.relVol)	1	Maximum relative element volume	Global
tpfmm.minqual	tpfmm.minOp(qual_spatial)		Minimum element quality	Global
tpfmm.FrR_mesh	d(r,R)	1	Mesh deformation gradient, rR component	Domains 1–2
tpfmm.FphiR_mesh	0	1	Mesh deformation gradient, phiR component	Domains 1–2
tpfmm.FzR_mesh	d(z,R)	1	Mesh deformation gradient, zR component	Domains 1–2
tpfmm.FrPHI_mesh	0	1	Mesh deformation	Domains 1–2

Name	Expression	Unit	Description	Selection
			gradient, rPHI component	
tpfmm.FphiPHI_mesh	1	1	Mesh deformation gradient, phiPHI component	Domains 1–2
tpfmm.FzPHI_mesh	0	1	Mesh deformation gradient, zPHI component	Domains 1–2
tpfmm.FrZ_mesh	d(r,Z)	1	Mesh deformation gradient, rZ component	Domains 1–2
tpfmm.FphiZ_mesh	0	1	Mesh deformation gradient, phiZ component	Domains 1–2
tpfmm.FzZ_mesh	d(z,Z)	1	Mesh deformation gradient, zZ component	Domains 1–2
tpfmm.J_mesh	tpfmm.FrR_mesh*tpfmm.FphiPHI_mesh*tpfmm.FzZ_mesh+tpfmm.FrPHI_mesh*tpfmm.FphiZ_mesh*tpfmm.FzR_mesh+tpfmm.FrZ_mesh*tpfmm.FphiR_mesh*tpfmm.FzPHI_mesh-tpfmm.FrR_mesh*tpfmm.FphiZ_mesh*tpfmm.FzPHI_mesh-tpfmm.FrPHI_mesh*tpfmm.FphiR_mesh*tpfmm.FzZ_mesh-tpfmm.FrZ_mesh*tpfmm.FphiPHI_mesh*tpfmm.FzR_mesh	1	Mesh deformation volume ratio	Domains 1–2
tpfmm.I1iso	-1.5+0.5*(tpfmm.FrR_mesh^2+tpfmm.FphiR_mesh^2+tpfmm.FzR_mesh^2+tpfmm.FrPHI_mesh^2+tpfmm.FphiPHI_m	1	First invariant of isochoric mesh strain tensor	Domains 1–2

Name	Expression	Unit	Description	Selection
	esh^2+tpfmm.FzPHI_mesh^2 +tpfmm.FrZ_mesh^2+tpfmm. FphiZ_mesh^2+tpfmm.FzZ_m esh^2)*tpfmm.J_mesh^(-2/3)			
tpfmm.I1isoMax	tpfmm.maxOp(tpfmm.I1iso)	1	Maximum element distortion	Global
tpfmm.PrR_mesh	d(tpfmm.FrR_mesh,TIME)	1/s	Mesh smoothing stress tensor, rR component	Domains 1–2
tpfmm.PphiR_mesh	d(tpfmm.FphiR_mesh,TIME)	1/s	Mesh smoothing stress tensor, phiR component	Domains 1–2
tpfmm.PzR_mesh	d(tpfmm.FzR_mesh,TIME)	1/s	Mesh smoothing stress tensor, zR component	Domains 1–2
tpfmm.PrPHI_mesh	d(tpfmm.FrPHI_mesh,TIME)	1/s	Mesh smoothing stress tensor, rPHI component	Domains 1–2
tpfmm.PphiPHI_mesh	d(tpfmm.FphiPHI_mesh,TIME)	1/s	Mesh smoothing stress tensor, phiPHI component	Domains 1–2
tpfmm.PzPHI_mesh	d(tpfmm.FzPHI_mesh,TIME)	1/s	Mesh smoothing stress tensor, zPHI component	Domains 1–2
tpfmm.PrZ_mesh	d(tpfmm.FrZ_mesh,TIME)	1/s	Mesh smoothing stress tensor, rZ component	Domains 1–2
tpfmm.PphiZ_mesh	d(tpfmm.FphiZ_mesh,TIME)	1/s	Mesh smoothing stress tensor,	Domains 1–2

Name	Expression	Unit	Description	Selection
			phiZ component	
tpfmm.PzZ_mesh	d(tpfmm.FzZ_mesh,TIME)	1/s	Mesh smoothing stress tensor, zZ component	Domains 1–2
tpfmm.PrR_test	test(tpfmm.FrR_mesh)	1	Mesh smoothing stress tensor, rR component	Domains 1–2
tpfmm.PphiR_test	test(tpfmm.FphiR_mesh)	1	Mesh smoothing stress tensor, phiR component	Domains 1–2
tpfmm.PzR_test	test(tpfmm.FzR_mesh)	1	Mesh smoothing stress tensor, zR component	Domains 1–2
tpfmm.PrPHI_test	test(tpfmm.FrPHI_mesh)	1	Mesh smoothing stress tensor, rPHI component	Domains 1–2
tpfmm.PphiPHI_test	test(tpfmm.FphiPHI_mesh)	1	Mesh smoothing stress tensor, phiPHI component	Domains 1–2
tpfmm.PzPHI_test	test(tpfmm.FzPHI_mesh)	1	Mesh smoothing stress tensor, zPHI component	Domains 1–2
tpfmm.PrZ_test	test(tpfmm.FrZ_mesh)	1	Mesh smoothing stress tensor, rZ component	Domains 1–2
tpfmm.PphiZ_test	test(tpfmm.FphiZ_mesh)	1	Mesh smoothing stress tensor,	Domains 1–2

Name	Expression	Unit	Description	Selection
			phiZ component	
tpfmm.PzZ_test	test(tpfmm.FzZ_mesh)	1	Mesh smoothing stress tensor, zZ component	Domains 1–2
tpfmm.G_mesh	1	1	Mesh tangent stiffness	Domains 1–2
tpfmm.vold1	tpfmm.intVolume1(2*pi*r)	m^3	Volume of domain	Global
tpfmm.vold2	tpfmm.intVolume2(2*pi*r)	m^3	Volume of domain	Global
rt	d(r,TIME)	m/s	Mesh velocity, r component	Global
phit	0	m/s	Mesh velocity, phi component	Global
zt	d(z,TIME)	m/s	Mesh velocity, z component	Global
tpfmm.pref	1[atm]	Pa	Reference pressure level	Domains 1–2
tpfmm.pA	tpfmm.pref+p	Pa	Absolute pressure	Domains 1–2

2.4.1 Free Deformation 1

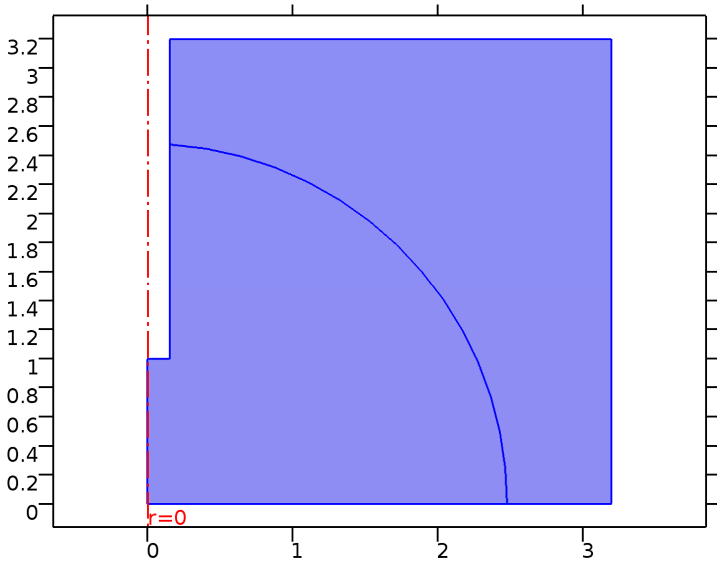

Free Deformation 1

Selection

Geometric entity level	Domain
Selection	Domains 1–2

Settings

Description	Value
Initial mesh displacement	{0, 0}

Shape functions

Name	Shape function	Unit	Description	Shape frame	Selection
r	Lagrange (Linear)	m	Spatial coordinate r	Spatial	Domains 1–2
z	Lagrange (Linear)	m	Spatial coordinate z	Spatial	Domains 1–2
r	Lagrange (Linear)	m	Spatial coordinate r	Material	Domains 1–2
z	Lagrange (Linear)	m	Spatial coordinate z	Material	Domains 1–2

Weak expressions

Weak expression	Integration frame	Selection
if(tpfmm.relVol<0,NaN*test(r),-Rr*test(rr)-Rz*test(rz)-Zr*test(zr)-Zz*test(zz))	Spatial	Domains 1–2

2.4.2 Prescribed Mesh Displacement 1

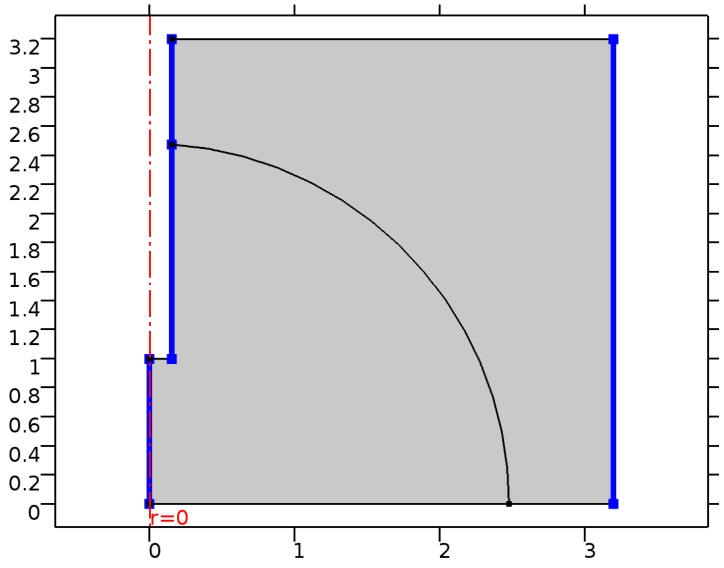

Prescribed Mesh Displacement 1

Selection

Geometric entity level	Boundary
Selection	Boundaries 1, 4–5, 8

Settings

Description	Value
Prescribed # displacement	{On, Off}
Prescribed mesh displacement	{0, 0}
Use weak constraints	Off

Variables

Name	Expression	Unit	Description	Selection
tpfmm.r_free	r	m	Coordinate, r component	Boundaries 1, 4–5, 8
tpfmm.z_free	z	m	Coordinate, z component	Boundaries 1, 4–5, 8

Shape functions

Constraint	Constraint force	Shape function	Selection
R-tpfmm.r_free	test(-tpfmm.r_free)	Lagrange (Linear)	Boundaries 1, 4–5, 8
0	0		Boundaries 1, 4–5, 8
0	test(-tpfmm.z_free)	Lagrange (Linear)	Boundaries 1, 4–5, 8

2.4.3 Axial Symmetry 1

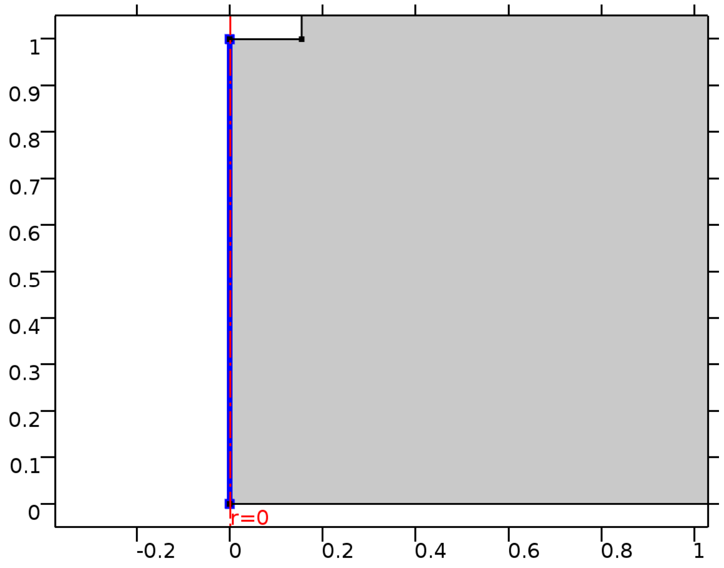

Axial Symmetry 1

Selection

Geometric entity level	Boundary
Selection	Boundary 1

2.4.4 Wall 1

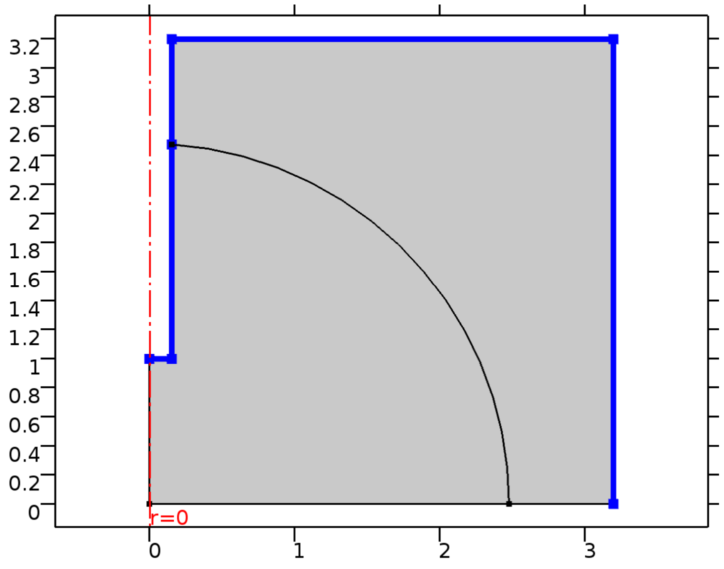

Wall 1

Selection

Geometric entity level	Boundary

Selection	Boundaries 3–6, 8

Equations

$$\mathbf{u} = \mathbf{0}$$

Settings

Description	Value
Temperature	User defined
Temperature	293.15[K]
Electric field	User defined
Electric field	{0, 0, 0}
Boundary condition	No slip
Apply reaction terms on	Individual dependent variables
Use weak constraints	Off
Constraint method	Elemental

Variables

Name	Expression	Unit	Description	Selection
tpfmm.ubndr	0	m/s	Velocity at boundary, r component	Boundaries 3–6, 8
tpfmm.ubndphi	0	m/s	Velocity at boundary, phi component	Boundaries 3–6, 8
tpfmm.ubndz	0	m/s	Velocity at boundary, z component	Boundaries 3–6, 8

Shape functions

Constraint	Constraint force	Shape function	Selection
-u+tpfmm.ubndr	test(-u)	Lagrange (Linear)	Boundaries 3–6, 8
0	0		Boundaries 3–6, 8
-w+tpfmm.ubndz	test(-w)	Lagrange (Linear)	Boundaries 3–6, 8

2.4.5 Fluid Properties 1

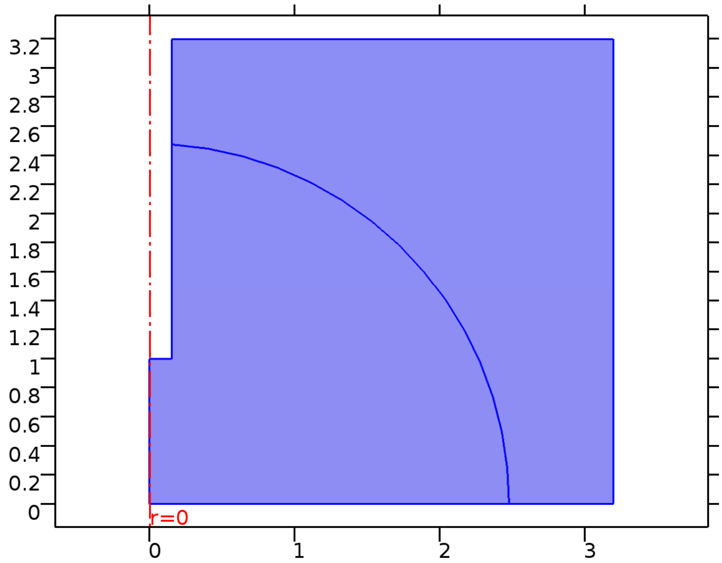

Fluid Properties 1

Selection

Geometric entity level	Domain
Selection	Domains 1–2

Equations

$$\rho\frac{\partial \mathbf{u}}{\partial t} + \rho(\mathbf{u}\cdot\nabla)\mathbf{u} = \nabla\cdot\left[-p\mathbf{I} + \mu\left(\nabla\mathbf{u} + (\nabla\mathbf{u})^T\right) - \frac{2}{3}\mu(\nabla\cdot\mathbf{u})\mathbf{I}\right] + \mathbf{F}$$

$$\frac{\partial\rho}{\partial t} + \nabla\cdot(\rho\mathbf{u}) = 0$$

Settings

Description	Value
Density	From material
Dynamic viscosity	From material
Reference length	1
Reference length scale	Automatic
Mixing length limit	Automatic

Properties from material

Property	Material	Property group
Density	Air	Basic

Property	Material	Property group
Dynamic viscosity	Air	Basic
Density	Water, liquid	Basic
Dynamic viscosity	Water, liquid	Basic

Variables

Name	Expression	Unit	Description	Selection
urt	root.comp1.urTIME-urr*d(r,TIME)-urz*d(z,TIME)	1/s^2	Gradient of u, first time derivative, r component	Domains 1–2
uzt	root.comp1.uzTIME-uzr*d(r,TIME)-uzz*d(z,TIME)	1/s^2	Gradient of u, first time derivative, z component	Domains 1–2
urtt	d(root.comp1.urTIME-urr*d(r,TIME)-urz*d(z,TIME),TIME)-d(root.comp1.urTIME-urr*d(r,TIME)-urz*d(z,TIME),r)*d(r,TIME)-d(root.comp1.urTIME-urr*d(r,TIME)-urz*d(z,TIME),z)*d(z,TIME)	1/s^3	Gradient of u, second time derivative, r component	Domains 1–2
uztt	d(root.comp1.uzTIME-uzr*d(r,TIME)-uzz*d(z,TIME),TIME)-d(root.comp1.uzTIME-uzr*d(r,TIME)-uzz*d(z,TIME),r)*d(r,TIME)-d(root.comp1.uzTIME-uzr*d(r,TIME)-uzz*d(z,TIME),z)*d(z,TIME)	1/s^3	Gradient of u, second time derivative, z component	Domains 1–2
wrt	root.comp1.wrTIME-wrr*d(r,TIME)-wrz*d(z,TIME)	1/s^2	Gradient of w, first time derivative, r component	Domains 1–2
wzt	root.comp1.wzTIME-wzr*d(r,TIME)-wzz*d(z,TIME)	1/s^2	Gradient of w, first time derivative, z component	Domains 1–2
wrtt	d(root.comp1.wrTIME-wrr*d(r,TIME)-	1/s^3	Gradient of w, second time	Domains 1–2

Name	Expression	Unit	Description	Selection
	wrz*d(z,TIME),TIME)-d(root.comp1.wrTIME-wrr*d(r,TIME)-wrz*d(z,TIME),r)*d(r,TIME)-d(root.comp1.wrTIME-wrr*d(r,TIME)-wrz*d(z,TIME),z)*d(z,TIME)		derivative, r component	
wztt	d(root.comp1.wzTIME-wzr*d(r,TIME)-wzz*d(z,TIME),TIME)-d(root.comp1.wzTIME-wzr*d(r,TIME)-wzz*d(z,TIME),r)*d(r,TIME)-d(root.comp1.wzTIME-wzr*d(r,TIME)-wzz*d(z,TIME),z)*d(z,TIME)	1/s^3	Gradient of w, second time derivative, z component	Domains 1–2
ut	root.comp1.uTIME-ur*d(r,TIME)-uz*d(z,TIME)	m/s^2	Velocity field, first time derivative, r component	Domains 1–2
wt	root.comp1.wTIME-wr*d(r,TIME)-wz*d(z,TIME)	m/s^2	Velocity field, first time derivative, z component	Domains 1–2
utt	d(root.comp1.uTIME-ur*d(r,TIME)-uz*d(z,TIME),TIME)-d(root.comp1.uTIME-ur*d(r,TIME)-uz*d(z,TIME),r)*d(r,TIME)-d(root.comp1.uTIME-ur*d(r,TIME)-uz*d(z,TIME),z)*d(z,TIME)	m/s^3	Velocity field, second time derivative, r component	Domains 1–2
wtt	d(root.comp1.wTIME-wr*d(r,TIME)-wz*d(z,TIME),TIME)-d(root.comp1.wTIME-wr*d(r,TIME)-wz*d(z,TIME),r)*d(r,TIME)-	m/s^3	Velocity field, second time derivative, z component	Domains 1–2

Name	Expression	Unit	Description	Selection
	d(root.comp1.wTIME-wr*d(r,TIME)-wz*d(z,TIME),z)*d(z,TIME)			
prt	root.comp1.prTIME-prr*d(r,TIME)-prz*d(z,TIME)	kg/(m^2*s^3)	Gradient of p, first time derivative, r component	Domains 1–2
pzt	root.comp1.pzTIME-pzr*d(r,TIME)-pzz*d(z,TIME)	kg/(m^2*s^3)	Gradient of p, first time derivative, z component	Domains 1–2
prtt	d(root.comp1.prTIME-prr*d(r,TIME)-prz*d(z,TIME),TIME)-d(root.comp1.prTIME-prr*d(r,TIME)-prz*d(z,TIME),r)*d(r,TIME)-d(root.comp1.prTIME-prr*d(r,TIME)-prz*d(z,TIME),z)*d(z,TIME)	kg/(m^2*s^4)	Gradient of p, second time derivative, r component	Domains 1–2
pztt	d(root.comp1.pzTIME-pzr*d(r,TIME)-pzz*d(z,TIME),TIME)-d(root.comp1.pzTIME-pzr*d(r,TIME)-pzz*d(z,TIME),r)*d(r,TIME)-d(root.comp1.pzTIME-pzr*d(r,TIME)-pzz*d(z,TIME),z)*d(z,TIME)	kg/(m^2*s^4)	Gradient of p, second time derivative, z component	Domains 1–2
pt	root.comp1.pTIME-pr*d(r,TIME)-pz*d(z,TIME)	Pa/s	Pressure, first time derivative	Domains 1–2
ptt	d(root.comp1.pTIME-pr*d(r,TIME)-pz*d(z,TIME),TIME)-d(root.comp1.pTIME-pr*d(r,TIME)-pz*d(z,TIME),r)*d(r,TIME)-d(root.comp1.pTIME-pr*d(r,TIME)-	Pa/s^2	Pressure, second time derivative	Domains 1–2

Name	Expression	Unit	Description	Selection
	pz*d(z,TIME),z)*d(z,TIME)			
tpfmm.rho	material.rho	kg/m^3	Density	Domains 1–2
tpfmm.mu	material.mu	Pa*s	Dynamic viscosity	Domains 1–2
tpfmm.divu	ur+if(abs(r)<0.001*h, ur,u/r)+wz	1/s	Divergence of velocity field	Domains 1–2
tpfmm.sr	sqrt(0.5*(4*ur^2+2*(uz+wr)^2+4*if(abs(r)<0.001*h,ur,u/r)^2+4*wz^2)+eps)	1/s	Shear rate	Domains 1–2
tpfmm.Fr	0	N/m^3	Volume force, r component	Domains 1–2
tpfmm.Fphi	0	N/m^3	Volume force, phi component	Domains 1–2
tpfmm.Fz	0	N/m^3	Volume force, z component	Domains 1–2
tpfmm.U	sqrt(u^2+w^2)	m/s	Velocity magnitude	Domains 1–2
tpfmm.vorticityr	0	1/s	Vorticity field, r component	Domains 1–2
tpfmm.vorticityphi	-wr+uz	1/s	Vorticity field, phi component	Domains 1–2
tpfmm.vorticityz	0	1/s	Vorticity field, z component	Domains 1–2
tpfmm.vort_magn	sqrt(tpfmm.vorticityr^2+tpfmm.vorticityphi^2+tpfmm.vorticityz^2)	1/s	Vorticity magnitude	Domains 1–2
tpfmm.cellRe	0.25*tpfmm.rho*sqrt(emetric_spatial(u-d(r,TIME),w-d(z,TIME))/emetric2_spatial)/tpfmm.mu	1	Cell Reynolds number	Domains 1–2
tpfmm.nu	tpfmm.mu/tpfmm.rho	m^2/s	Kinematic viscosity	Domains 1–2
tpfmm.betaT	d(tpfmm.rho,p)/tpfmm.rho	1/Pa	Isothermal compressibility coefficient	Domains 1–2
tpfmm.T_stressr	2*tpfmm.mu*ur*tpfmm.nrmesh+tpfmm.	N/m^2	Total stress, r component	Boundaries 2–9

Name	Expression	Unit	Description	Selection
	mu*(uz+wr)*tpfmm.nzmesh-2*tpfmm.divu*tpfmm.mu*tpfmm.nrmesh/3-p*tpfmm.nrmesh			
tpfmm.T_stressphi	tpfmm.nphimesh*(2*tpfmm.mu*if(abs(r)<0.001*h,ur,u/r)-2*tpfmm.divu*tpfmm.mu/3-p)	N/m^2	Total stress, phi component	Boundaries 2–9
tpfmm.T_stressz	tpfmm.mu*(wr+uz)*tpfmm.nrmesh+2*tpfmm.mu*wz*tpfmm.nzmesh-2*tpfmm.divu*tpfmm.mu*tpfmm.nzmesh/3-p*tpfmm.nzmesh	N/m^2	Total stress, z component	Boundaries 2–9
tpfmm.K_stressr	tpfmm.mu*(2*ur*tpfmm.nrmesh+(uz+wr)*tpfmm.nzmesh-2*tpfmm.divu*tpfmm.nrmesh/3)	N/m^2	Viscous stress, r component	Boundaries 2–9
tpfmm.K_stressphi	tpfmm.mu*tpfmm.nphimesh*(2*if(abs(r)<0.001*h,ur,u/r)-2*tpfmm.divu/3)	N/m^2	Viscous stress, phi component	Boundaries 2–9
tpfmm.K_stressz	tpfmm.mu*((wr+uz)*tpfmm.nrmesh+2*wz*tpfmm.nzmesh-2*tpfmm.divu*tpfmm.nzmesh/3)	N/m^2	Viscous stress, z component	Boundaries 2–9
tpfmm.upwind_helpr	u-d(r,TIME)	m/s	Upwind term, r component	Domains 1–2
tpfmm.upwind_helpphi	0	m/s	Upwind term, phi component	Domains 1–2
tpfmm.upwind_helpz	w-d(z,TIME)	m/s	Upwind term, z component	Domains 1–2
tpfmm.K_stress_tensorrr	tpfmm.mu*(2*ur-2*tpfmm.divu/3)	N/m^2	Viscous stress tensor, rr component	Domains 1–2

Name	Expression	Unit	Description	Selection
tpfmm.K_stress_tensorphir	0	N/m^2	Viscous stress tensor, phir component	Domains 1–2
tpfmm.K_stress_tensorzr	tpfmm.mu*(wr+uz)	N/m^2	Viscous stress tensor, zr component	Domains 1–2
tpfmm.K_stress_tensorrphi	0	N/m^2	Viscous stress tensor, rphi component	Domains 1–2
tpfmm.K_stress_tensorphiphi	tpfmm.mu*(2*if(abs(r)<0.001*h,ur,u/r)-2*tpfmm.divu/3)	N/m^2	Viscous stress tensor, phiphi component	Domains 1–2
tpfmm.K_stress_tensorzphi	0	N/m^2	Viscous stress tensor, zphi component	Domains 1–2
tpfmm.K_stress_tensorrz	tpfmm.mu*(uz+wr)	N/m^2	Viscous stress tensor, rz component	Domains 1–2
tpfmm.K_stress_tensorphiz	0	N/m^2	Viscous stress tensor, phiz component	Domains 1–2
tpfmm.K_stress_tensorzz	tpfmm.mu*(2*wz-2*tpfmm.divu/3)	N/m^2	Viscous stress tensor, zz component	Domains 1–2
tpfmm.K_stress_tensor_testrr	tpfmm.mu*(2*test(ur)-2*(test(ur)+if(abs(r)<0.001*h,test(ur),test(u)/r)+test(wz))/3)	N/m^2	Viscous stress tensor test, rr component	Domains 1–2
tpfmm.K_stress_tensor_testphir	0	N/m^2	Viscous stress tensor test, phir component	Domains 1–2
tpfmm.K_stress_tensor_testzr	tpfmm.mu*(test(wr)+test(uz))	N/m^2	Viscous stress tensor test, zr component	Domains 1–2
tpfmm.K_stress_tensor_testrphi	0	N/m^2	Viscous stress tensor test, rphi component	Domains 1–2
tpfmm.K_stress_tensor_testphiphi	tpfmm.mu*(2*if(abs(r)<0.001*h,test(ur),test(u)/r)-	N/m^2	Viscous stress tensor test, phiphi component	Domains 1–2

Name	Expression	Unit	Description	Selection
	2*(test(ur)+if(abs(r)<0.001*h,test(ur),test(u)/r)+test(wz))/3)			
tpfmm.K_stress_tensor_testzphi	0	N/m^2	Viscous stress tensor test, zphi component	Domains 1–2
tpfmm.K_stress_tensor_testrz	tpfmm.mu*(test(uz)+test(wr))	N/m^2	Viscous stress tensor test, rz component	Domains 1–2
tpfmm.K_stress_tensor_testphiz	0	N/m^2	Viscous stress tensor test, phiz component	Domains 1–2
tpfmm.K_stress_tensor_testzz	tpfmm.mu*(2*test(wz)-2*(test(ur)+if(abs(r)<0.001*h,test(ur),test(u)/r)+test(wz))/3)	N/m^2	Viscous stress tensor test, zz component	Domains 1–2
tpfmm.Qvd	tpfmm.mu*((2*ur-2*(ur+if(abs(r)<0.001*h,ur,u/r)+wz)/3)*ur+(uz+wr)*uz+(2*if(abs(r)<0.001*h,ur,u/r)-2*(ur+if(abs(r)<0.001*h,ur,u/r)+wz)/3)*if(abs(r)<0.001*h,ur,u/r)+(uz+wr)*wr+(2*wz-2*(ur+if(abs(r)<0.001*h,ur,u/r)+wz)/3)*wz)	W/m^3	Viscous dissipation	Domains 1–2
tpfmm.res_u	tpfmm.rho*(root.comp1.uTIME-d(u,r)*d(r,TIME)-d(u,z)*d(z,TIME))+pr+tpfmm.rho*u*ur+tpfmm.rho*w*uz-(d(2*ur-2*tpfmm.divu/3,r)+if(abs(r)<0.001*h,d(2*ur-2*tpfmm.divu/3,r),(2*ur-2*tpfmm.divu/3)/r)+d(uz+wr,z)-(2*if(abs(r)<0.001*h,ur,u/r)-	N/m^3	Equation residual	Domains 1–2

Name	Expression	Unit	Description	Selection
	2*tpfmm.divu/3)/r)*tpfmm.mu-tpfmm.Fr			
tpfmm.res_v	-tpfmm.Fphi	N/m^3	Equation residual	Domains 1–2
tpfmm.res_w	tpfmm.rho*(root.comp1.wTIME-d(w,r)*d(r,TIME)-d(w,z)*d(z,TIME))+tpfmm.rho*u*wr+pz+tpfmm.rho*w*wz-(d(wr+uz,r)+if(abs(r)<0.001*h,d(wr+uz,r),(wr+uz)/r)+d(2*wz-2*tpfmm.divu/3,z))*tpfmm.mu-tpfmm.Fz	N/m^3	Equation residual	Domains 1–2
tpfmm.res_p	d(tpfmm.rho,TIME)-d(tpfmm.rho,r)*d(r,TIME)-d(tpfmm.rho,z)*d(z,TIME)+tpfmm.rho*tpfmm.divu+u*d(tpfmm.rho,r)+w*d(tpfmm.rho,z)	kg/(m^3*s)	Pressure equation residual	Domains 1–2

Shape functions

Name	Shape function	Unit	Description	Shape frame	Selection
u	Lagrange (Linear)	m/s	Velocity field, r component	Spatial	Domains 1–2
w	Lagrange (Linear)	m/s	Velocity field, z component	Spatial	Domains 1–2
p	Lagrange (Linear)	Pa	Pressure	Spatial	Domains 1–2

Weak expressions

Weak expression	Integration frame	Selection
-2*tpfmm.rho*((root.comp1.uTIME-ur*d(r,TIME)-uz*d(z,TIME))*test(u)+(root.comp1.wTIME-wr*d(r,TIME)-wz*d(z,TIME))*test(w))*pi*r	Spatial	Domains 1–2
2*((p-tpfmm.K_stress_tensorrr)*test(ur)-tpfmm.K_stress_tensorrz*test(uz)+(p-tpfmm.K_stress_tensorphiphi)*if(abs(r)<0.001*h,test(ur),te	Spatial	Domains 1–2

Weak expression	Integration frame	Selection
st(u)/r)-tpfmm.K_stress_tensorzr*test(wr)+(p-tpfmm.K_stress_tensorzz)*test(wz))*pi*r		
2*(tpfmm.Fr*test(u)+tpfmm.Fz*test(w)-tpfmm.rho*(ur*u+uz*w)*test(u)-tpfmm.rho*(wr*u+wz*w)*test(w))*pi*r	Spatial	Domains 1–2
2*test(p)*(-tpfmm.rho*tpfmm.divu-u*d(tpfmm.rho,r)-w*d(tpfmm.rho,z)-d(tpfmm.rho,TIME)+d(tpfmm.rho,r)*d(r,TIME)+d(tpfmm.rho,z)*d(z,TIME))*pi*r	Spatial	Domains 1–2
2*tpfmm.streamlinens*pi*r	Spatial	Domains 1–2

2.4.6 Initial Values 1

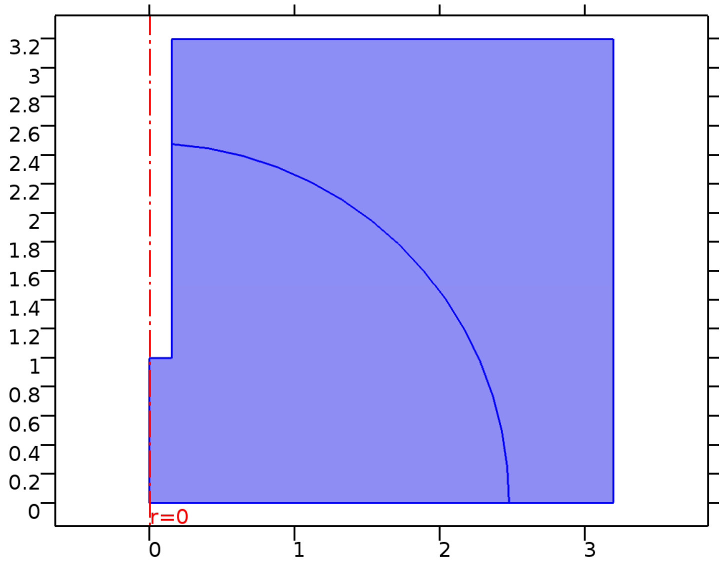

Initial Values 1

Selection

Geometric entity level	Domain
Selection	Domains 1–2

Settings

Description	Value
Velocity field	{0, 0, 0}
Pressure	0

2.4.7 Fluid-Fluid Interface 1

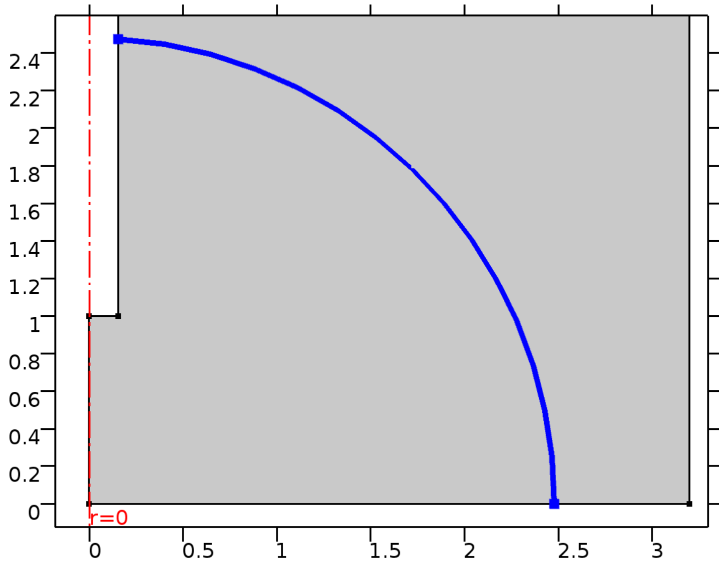

Fluid-Fluid Interface 1

Selection

Geometric entity level	Boundary
Selection	Boundary 9

Equations

$$\mathbf{u}_1 = \mathbf{u}_2, \quad \mathbf{n}_1 \cdot \mathbf{T}_1 - \mathbf{n}_1 \cdot \mathbf{T}_2 = \sigma(\nabla_t \cdot \mathbf{n}_1)\mathbf{n}_1 - \nabla_t \sigma$$

$$\mathbf{u}_1 = \mathbf{u}_2 + M_f\left(\frac{1}{\rho_1} + \frac{1}{\rho_2}\right)\mathbf{n}_1$$

$$\mathbf{u}_{mesh} = (\mathbf{u}_1 \cdot \mathbf{n}_1) - \frac{M_f}{\rho_1}\mathbf{n}_1$$

Settings

Description	Value
Mass flux	User defined
Mass flux	0
Surface tension coefficient	gamma
Surface tension coefficient	User defined

Variables

Name	Expression	Unit	Description	Selection
tpfmm.r_free	r	m	Coordinate, r component	Boundary 9

Name	Expression	Unit	Description	Selection
tpfmm.z_free	z	m	Coordinate, z component	Boundary 9
tpfmm.ffi1.input.Mf	0	kg/(m^2*s)	Mass flux	Global
tpfmm.sigma	gamma	N/m	Surface tension coefficient	Boundary 9
tpfmm.rt_free	rTIME	m/s	Mesh velocity, r component	Boundary 9
tpfmm.phit_free	0	m/s	Mesh velocity, phi component	Boundary 9
tpfmm.zt_free	zTIME	m/s	Mesh velocity, z component	Boundary 9

Shape functions

Name	Shape function	Unit	Description	Shape frame	Selection
u	Lagrange (Linear)	m/s	Velocity field, r component	Spatial	Boundary 9
w	Lagrange (Linear)	m/s	Velocity field, z component	Spatial	Boundary 9
p	Lagrange (Linear)	Pa	Pressure	Spatial	Boundary 9
r_lm	Lagrange (Linear)	1	Lagrange multiplier for spatial coordinate r	Spatial	Boundary 9
z_lm	Lagrange (Linear)	1	Lagrange multiplier for spatial coordinate z	Spatial	Boundary 9

Weak expressions

Weak expression	Integration frame	Selection
-2*((1-tpfmm.nr^2)*down(test(ur))-2*tpfmm.nr*tpfmm.nphi*down(0)-tpfmm.nr*tpfmm.nz*down(test(uz))+(1-tpfmm.nphi^2)*down(if(abs(r)<0.001*h,test(ur),test(u)/r))-2*tpfmm.nphi*tpfmm.nz*down(0)-tpfmm.nz*tpfmm.nr*down(test(wr))+(1-tpfmm.nz^2)*down(test(wz)))*tpfmm.sigma*pi*r	Spatial	Boundary 9
(tpfmm.rt_free*tpfmm.nr+tpfmm.phit_free*tpfmm.nphi+tpfmm.zt_free*tpfmm.nz-(down(u)-tpfmm.ffi1.input.Mf*tpfmm.nr/down(tpfmm.rho))*tpfmm.n	Spatial	Boundary 9

Weak expression	Integration frame	Selection
r-(down(0)-tpfmm.ffi1.input.Mf*tpfmm.nphi/down(tpfmm.rho))*tpfmm.nphi-(down(w)-tpfmm.ffi1.input.Mf*tpfmm.nz/down(tpfmm.rho))*tpfmm.nz)*test(-r_lm)		
-test(tpfmm.nr*tpfmm.r_free+tpfmm.nz*tpfmm.z_free)*r_lm	Spatial	Boundary 9

Shape functions

Constraint	Constraint force	Shape function	Selection
down(u)-up(u)-tpfmm.ffi1.input.Mf*tpfmm.nr*(1/down(tpfmm.rho)-1/up(tpfmm.rho))	test(down(u)-up(u))	Lagrange (Linear)	Boundary 9
down(0)-up(0)-tpfmm.ffi1.input.Mf*tpfmm.nphi*(1/down(tpfmm.rho)-1/up(tpfmm.rho))	test(down(0)-up(0))		Boundary 9
down(w)-up(w)-tpfmm.ffi1.input.Mf*tpfmm.nz*(1/down(tpfmm.rho)-1/up(tpfmm.rho))	test(down(w)-up(w))	Lagrange (Linear)	Boundary 9

Wall-Fluid Interface 1

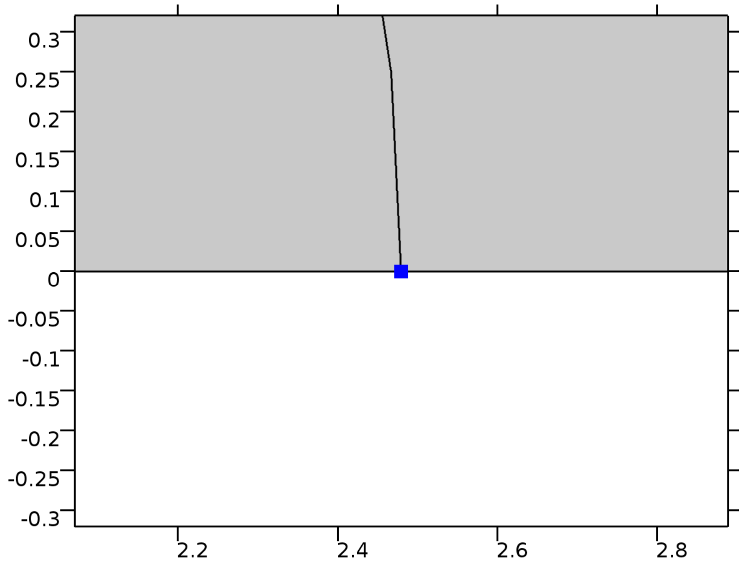

Wall-Fluid Interface 1

Selection

Geometric entity level	Point
Selection	Point 6

Equations

$$\theta_c = \theta_w$$

Settings

Description	Value
Contact angle	theta
Specify contact angle	Directly

Variables

Name	Expression	Unit	Description	Selection
tpfmm.iNr	tpfmm.nr		Interface normal, r component	Boundary 9
tpfmm.iNphi	tpfmm.nphi		Interface normal, phi component	Boundary 9
tpfmm.iNz	tpfmm.nz		Interface normal, z component	Boundary 9
tpfmm.sNr	tpfmm.nr		Surface normal, r component	Boundaries 2, 7
tpfmm.sNphi	tpfmm.nphi		Surface normal, phi component	Boundaries 2, 7
tpfmm.sNz	tpfmm.nz		Surface normal, z component	Boundaries 2, 7
tpfmm.thetac	theta	rad	Contact angle	Point 6
tpfmm.tcr	(tpfmm.sNz*tpfmm.iNphi-tpfmm.sNphi*tpfmm.iNz)/sqrt((tpfmm.sNz*tpfmm.iNphi-tpfmm.sNphi*tpfmm.iNz)^2+(-tpfmm.sNz*tpfmm.iNr+tpfmm.sNr*tpfmm.iNz)^2+(tpfmm.sNphi*tpfmm.iNr-tpfmm.sNr*tpfmm.iNphi)^2+eps)		Contact tangent, r component	Point 6

Name	Expression	Unit	Description	Selection
tpfmm.tcphi	(-tpfmm.sNz*tpfmm.iNr+tpfmm.sNr*tpfmm.iNz)/sqrt((tpfmm.sNz*tpfmm.iNphi-tpfmm.sNphi*tpfmm.iNz)^2+(-tpfmm.sNz*tpfmm.iNr+tpfmm.sNr*tpfmm.iNz)^2+(tpfmm.sNphi*tpfmm.iNr-tpfmm.sNr*tpfmm.iNphi)^2+eps)		Contact tangent, phi component	Point 6
tpfmm.tcz	(tpfmm.sNphi*tpfmm.iNr-tpfmm.sNr*tpfmm.iNphi)/sqrt((tpfmm.sNz*tpfmm.iNphi-tpfmm.sNphi*tpfmm.iNz)^2+(-tpfmm.sNz*tpfmm.iNr+tpfmm.sNr*tpfmm.iNz)^2+(tpfmm.sNphi*tpfmm.iNr-tpfmm.sNr*tpfmm.iNphi)^2+eps)		Contact tangent, z component	Point 6
tpfmm.mir	tpfmm.iNz*tpfmm.tcphi-tpfmm.iNphi*tpfmm.tcz		Interface binormal, r component	Point 6
tpfmm.miphi	-tpfmm.iNz*tpfmm.tcr+tpfmm.iNr*tpfmm.tcz		Interface binormal, phi component	Point 6
tpfmm.miz	tpfmm.iNphi*tpfmm.tcr-tpfmm.iNr*tpfmm.tcphi		Interface binormal, z component	Point 6
tpfmm.msr	tpfmm.sNz*tpfmm.tcphi-tpfmm.sNphi*tpfmm.tcz		Surface binormal, r component	Point 6
tpfmm.msphi	-tpfmm.sNz*tpfmm.tcr+tpfmm.sNr*tpfmm.tcz		Surface binormal, phi component	Point 6
tpfmm.msz	tpfmm.sNphi*tpfmm.tcr-tpfmm.sNr*tpfmm.tcphi		Surface binormal, z component	Point 6

Weak expressions

Weak expression	Integration frame	Selection
2*tpfmm.sigma*cos(tpfmm.thetac)*(test(u)*(-tpfmm.sNz*tpfmm.tcphi+tpfmm.sNphi*tpfmm.tcz)+test(w)*(-tpfmm.sNphi*tpfmm.tcr+tpfmm.sNr*tpfmm.tcphi))*pi*r	Spatial	Point 6

Shape functions

Constraint	Constraint force	Shape function	Selection
-u*tpfmm.sNr-w*tpfmm.sNz	-test(u)*tpfmm.sNr-test(w)*tpfmm.sNz	Lagrange (Linear)	Point 6

2.4.8 Prescribed Mesh Displacement 2

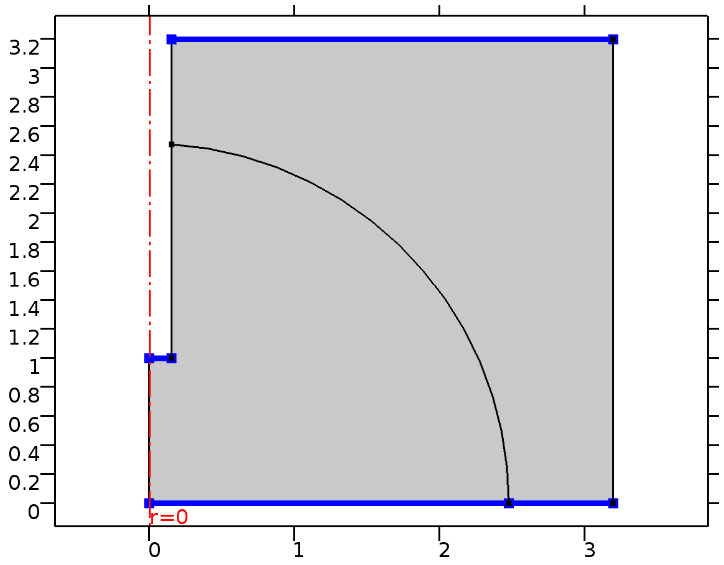

Prescribed Mesh Displacement 2

Selection

Geometric entity level	Boundary
Selection	Boundaries 2–3, 6–7

Settings

Description	Value
Prescribed # displacement	{Off, On}
Use weak constraints	Off

Variables

Name	Expression	Unit	Description	Selection
tpfmm.r_free	r	m	Coordinate, r component	Boundaries 2–3, 6–7
tpfmm.z_free	z	m	Coordinate, z component	Boundaries 2–3, 6–7

Shape functions

Constraint	Constraint force	Shape function	Selection
0	test(-tpfmm.r_free)	Lagrange (Linear)	Boundaries 2–3, 6–7

Constraint	Constraint force	Shape function	Selection
0	0		Boundaries 2–3, 6–7
Z-tpfmm.z_free	test(-tpfmm.z_free)	Lagrange (Linear)	Boundaries 2–3, 6–7

2.4.9 Pressure Point Constraint 1

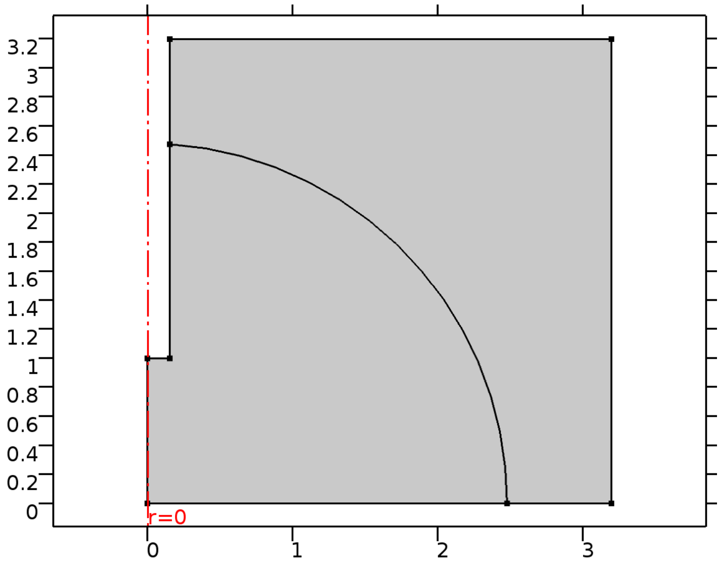

Pressure Point Constraint 1

Selection

Geometric entity level	Point
Selection	No points

Settings

Description	Value
Pressure	0
Apply reaction terms on	All physics (symmetric)
Use weak constraints	Off
Constraint method	Elemental

2.4.10 Navier Slip 1

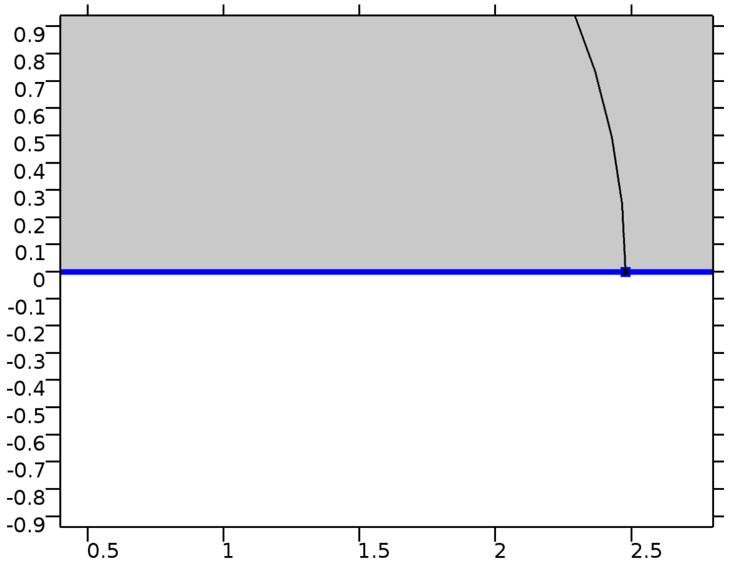

Navier Slip 1

Selection

Geometric entity level	Boundary
Selection	Boundaries 2, 7

Equations

$$\mathbf{u}\cdot\mathbf{n}=0,\quad \mathbf{n}\cdot T=-\frac{\mu}{\beta}\mathbf{u}$$

Settings

Description	Value
Slip length	0.2*h
Boundary condition	Stationary wall

Variables

Name	Expression	Unit	Description	Selection
tpfmm.beta	0.2*h	m	Slip length	Boundaries 2, 7

Weak expressions

Weak expression	Integration frame	Selection
-2*(u*test(u)+w*test(w))*tpfmm.mu*pi*r/tpfmm.beta	Spatial	Boundaries 2, 7

Shape functions

Constraint	Constraint force	Shape function	Selection
-tpfmm.nr*u-tpfmm.nz*w	-tpfmm.nr*test(u)-tpfmm.nz*test(w)	Lagrange (Linear)	Boundaries 2, 7

2.5 Mesh 1

Mesh statistics

Description	Value
Minimum element quality	0.7437
Average element quality	0.9844
Triangular elements	15422
Edge elements	400
Vertex elements	8

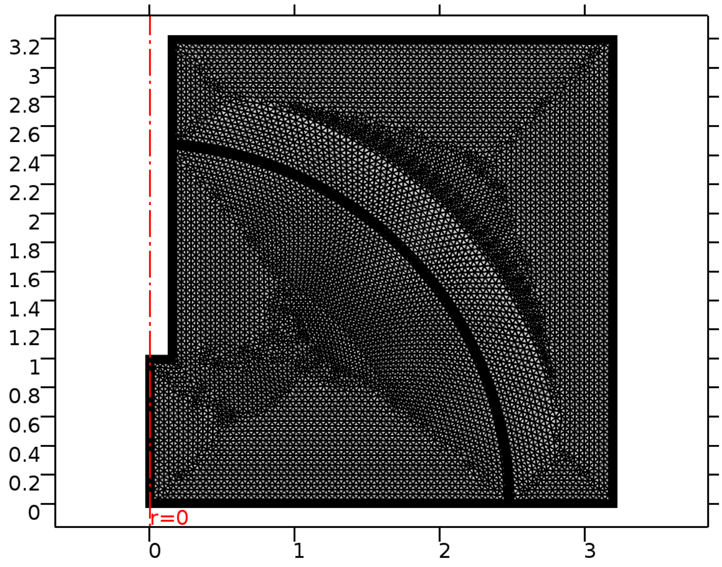

Mesh 1

2.5.1 Size (size)

Settings

Description	Value
Calibrate for	Fluid dynamics
Maximum element size	0.0416
Minimum element size	4.8E-4
Curvature factor	0.25

Description	Value
Maximum element growth rate	1.08
Predefined size	Extra fine

2.5.2 Size 1 (size1)

Selection

Geometric entity level	Boundary
Selection	Boundaries 3–6, 8

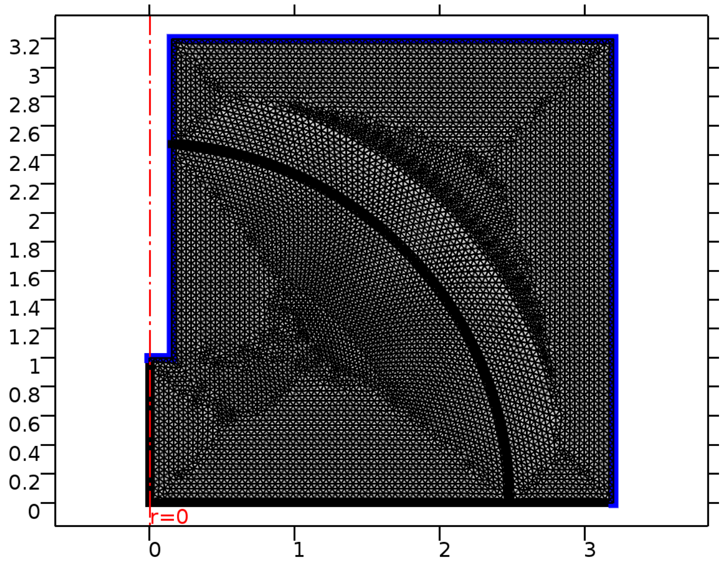

Size 1

Settings

Description	Value
Calibrate for	Fluid dynamics
Maximum element size	0.112
Minimum element size	0.0032
Curvature factor	0.3
Maximum element growth rate	1.13
Predefined size	Fine

2.5.3 Free Triangular 1 (ftri1)

Selection

Geometric entity level	Remaining

3 Study

Computation information

Computation time	3 min 18 s
CPU	Intel(R) Xeon(R) CPU E5-2670 0 @ 2.60GHz, 3 cores
Operating system	Windows 7

3.1 Phase Initialization

Study settings

Description	Value
Include geometric nonlinearity	Off

Physics and variables selection

Physics interface	Discretization
Laminar Two-Phase Flow, Moving Mesh (tpfmm)	physics

Mesh selection

Geometry	Mesh
Geometry 1 (geom1)	mesh1

3.2 Time Dependent

Study settings

Description	Value
Include geometric nonlinearity	Off

Times	Unit
range(0,1,57)	s

Physics and variables selection

Physics interface	Discretization
Laminar Two-Phase Flow, Moving Mesh (tpfmm)	physics

Mesh selection

Geometry	Mesh
Geometry 1 (geom1)	mesh1

3.3 Solver Configurations

3.3.1 Solution 2

Compile Equations: Phase Initialization (st1)

Study and step

Description	Value
Use study	Study
Use study step	Phase Initialization

Dependent Variables 1 (v1)

General

Description	Value
Defined by study step	Phase Initialization

Initial values of variables solved for

Description	Value
Solution	Zero

Values of variables not solved for

Description	Value
Solution	Zero

Spatial coordinates (Spatial) (comp1.rz) (comp1_rz)

General

Description	Value
Field components	{r, z}
Solve for this field	Off

Pressure (comp1.p) (comp1_p)

General

Description	Value
Field components	comp1.p
Solve for this field	Off

Lagrange multiplier for spatial coordinates (spatial) (Spatial) (comp1.rz_lm) (comp1_rz_lm)

General

Description	Value
Field components	{r_lm, z_lm}
Solve for this field	Off

Velocity field (Spatial) (comp1.u) (comp1_u)

General

Description	Value
Field components	{comp1.u, comp1.w}
Solve for this field	Off

Stationary Solver 1 (s1)

General

Description	Value
Defined by study step	Phase Initialization

Log

```
Stationary Solver 1 in Solution 2 started at 13-Feb-2018 16:13:52.
Linear solver
Number of degrees of freedom solved for: 0.
Symmetric matrices found.
Scales for dependent variables:
Orthonormal null-space function used.
Iter       SolEst      Damping     Stepsize #Res #Jac #Sol    LinErr    LinRes
   1            0    1.0000000          0    1    1    1         0         0
Stationary Solver 1 in Solution 2: Solution time: 3 s
                                   Physical memory: 743 MB
                                   Virtual memory: 921 MB
```

Fully Coupled (fcDef)

General

Description	Value
Linear solver	Direct

Solution Store 1 (su1)

General

Description	Value
Solution	Solution Store 1

Compile Equations: Time Dependent (st2)

Study and step

Description	Value
Use study	Study
Use study step	Time Dependent

Dependent Variables 2 (v2)

General

Description	Value
Defined by study step	Time Dependent

Initial values of variables solved for

Description	Value
Method	Solution
Solution	Solution 2

Values of variables not solved for

Description	Value
Method	Solution
Solution	Solution 2

Spatial coordinates (Spatial) (comp1.rz) (comp1_rz)

General

Description	Value
Field components	{r, z}

Pressure (comp1.p) (comp1_p)

General

Description	Value
Field components	comp1.p

Lagrange multiplier for spatial coordinates (spatial) (Spatial) (comp1.rz_lm) (comp1_rz_lm)

General

Description	Value
Field components	{r_lm, z_lm}

Velocity field (Spatial) (comp1.u) (comp1_u)

General

Description	Value
Field components	{comp1.u, comp1.w}

Time-Dependent Solver 1 (t1)

General

Description	Value
Defined by study step	Time Dependent
Time	{0, 1, 2, 3, 4, 5, 6, 7, 8, 9, 10, 11, 12, 13, 14, 15, 16, 17, 18, 19, 20, 21, 22, 23, 24, 25, 26, 27, 28, 29, 30, 31, 32, 33, 34, 35, 36, 37, 38, 39, 40, 41, 42, 43, 44, 45, 46, 47, 48, 49, 50, 51, 52, 53, 54, 55, 56, 57}

Absolute tolerance

Description	Value
Global method	Unscaled

Time stepping

Description	Value
Maximum BDF order	2
Error estimation	Exclude algebraic

Log

```
Time-Dependent Solver 1 in Solution 2 started at 13-Feb-2018 16:14:01.
Time-dependent solver (BDF)
Number of degrees of freedom solved for: 39699.
Nonsymmetric matrix found.
Scales for dependent variables:
Spatial coordinates (Spatial) (comp1.rz): 0.0032
Pressure (comp1.p): 1.9e+002
Lagrange multiplier for spatial coordinates (spatial) (Spatial) (comp1.rz_lm): 16
Velocity field (Spatial) (comp1.u): 0.92
Step        Time    Stepsize      Res  Jac  Sol Order Tfail NLfail   LinErr   LinRes
   0           0           - out    4    5    4                 0 7.7e-013 7.8e-015
   1    0.004573    0.004573        6    7    6     1     0      0 1.7e-009 5.7e-014
   2    0.009146    0.004573        8    9    8     1     0      0 1.1e-009 1.2e-013
   3    0.018292    0.009146       10   11   10     2     0      0 1.4e-010 1.3e-013
   4    0.036584    0.018292       12   13   12     1     0      0 1.5e-008 1.4e-013
   5    0.073168    0.036584       14   15   14     1     0      0   1e-008 3.4e-013
   6     0.14634    0.073168       16   17   16     1     0      0 7.6e-010 3.5e-013
   7     0.29267     0.14634       17   18   17     1     0      0 2.2e-008 1.7e-012
   8     0.58534     0.29267       19   20   19     1     0      0 6.9e-008 8.9e-013
   -           1           - out
   9      1.1707     0.58534       21   22   21     1     0      0   6e-008 5.1e-013
   -           2           - out
  10      2.3414      1.1707       23   24   23     1     0      0 3.4e-007 2.2e-013
   -           3           - out
   -           4           - out
  11      4.6827      2.3414       25   26   25     1     0      0 3.6e-006 7.2e-013
   -           5           - out
   -           6           - out
   -           7           - out
   -           8           - out
   -           9           - out
  12      9.3655      4.6827       27   28   27     1     0      0 3.5e-006 2.9e-012
   -          10           - out
   -          11           - out
   -          12           - out
   -          13           - out
   -          14           - out
   -          15           - out
  13      15.065         5.7       29   30   29     1     0      0 1.6e-006 2.4e-011
   -          16           - out
   -          17           - out
   -          18           - out
   -          19           - out
   -          20           - out
  14      20.765         5.7       31   32   31     1     0      0 4.8e-006 3.6e-012
   -          21           - out
   -          22           - out
   -          23           - out
   -          24           - out
   -          25           - out
   -          26           - out
  15      26.465         5.7       33   34   33     1     0      0 3.2e-007 1.2e-011
   -          27           - out
   -          28           - out
   -          29           - out
   -          30           - out
```

```
   -         31            - out
   -         32            - out
  16     32.165          5.7     35   36   35     1     0     0 2.3e-006 1.4e-011
   -         33            - out
   -         34            - out
   -         35            - out
   -         36            - out
   -         37            - out
  17     37.865          5.7     37   38   37     2     0     0 3.5e-006 2.8e-012
   -         38            - out
   -         39            - out
   -         40            - out
   -         41            - out
   -         42            - out
   -         43            - out
  18     43.565          5.7     39   40   39     2     0     0 1.8e-006 6.9e-012
   -         44            - out
   -         45            - out
   -         46            - out
   -         47            - out
   -         48            - out
   -         49            - out
  19     49.265          5.7     41   42   41     1     0     0 2.4e-006 1.7e-011
   -         50            - out
  20      50.69        1.425     51   51   50     1     0     1 2.2e-007 1.2e-012
   -         51            - out
   -         52            - out
   -         53            - out
  21      53.54         2.85     53   53   52     1     0     1 2.4e-006 3.1e-012
   -         54            - out
  22     54.965        1.425     57   56   55     1     0     2 2.9e-007 5.1e-012
   -         55            - out
   -         56            - out
  23         57       2.0345 out 61   60   59     1     0     2 2.3e-007   6e-013
Time-stepping completed.
Time-Dependent Solver 1 in Solution 2: Solution time: 182 s (3 minutes, 2 seconds)
                                       Physical memory: 902 MB
                                       Virtual memory: 1222 MB
```

Fully Coupled 1 (fc1)

General

Description	Value
Linear solver	Direct 1

Method and termination

Description	Value
Jacobian update	On every iteration
Maximum number of iterations	8

Direct 1 (d1)

General

Description	Value
Solver	PARDISO

4 Results

4.1 Data Sets

4.1.1 Study/Solution 2

Solution

Description	Value
Solution	Solution 2
Component	Save Point Geometry 1

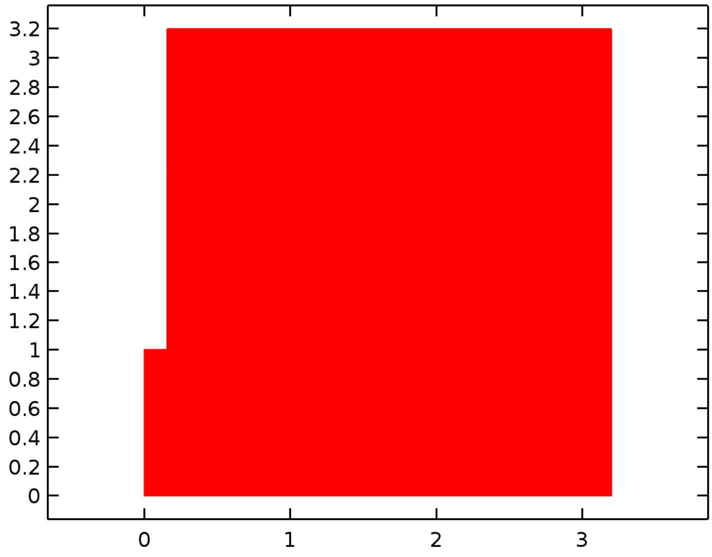

Data set: Study/Solution 2

4.1.2 Revolution 2D 2

Data

Description	Value
Data set	Study/Solution 2

Axis data

Description	Value
Axis entry method	Two points
Points	{{0, 0}, {0, 1}}

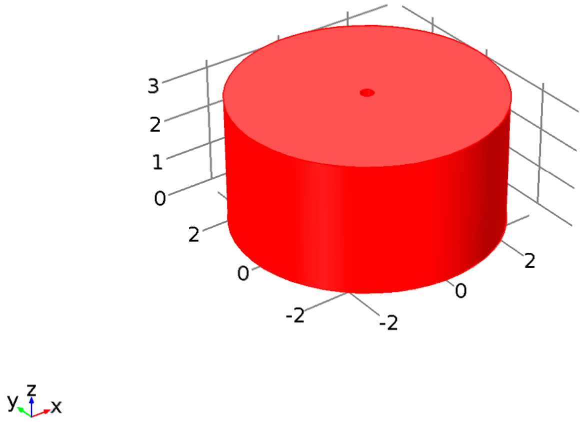

Data set: Revolution 2D 2

4.1.3 Revolution 2D 3

Data

Description	Value
Data set	Study/Solution 2

Axis data

Description	Value
Axis entry method	Two points
Points	{{0, 0}, {0, 1}}

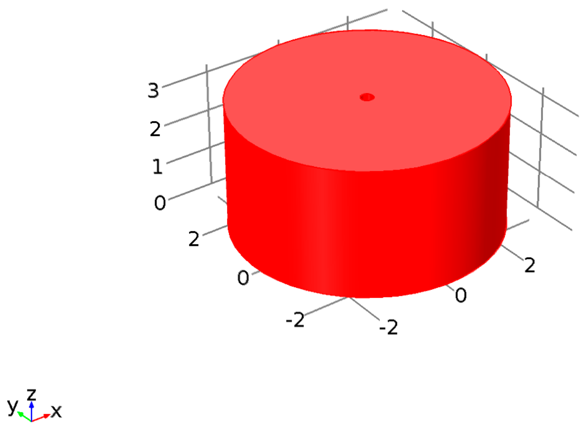

Data set: Revolution 2D 3

4.1.4 Revolution 2D 4

Data

Description	Value
Data set	Study/Solution 2

Axis data

Description	Value
Axis entry method	Two points
Points	{{0, 0}, {0, 1}}

Revolution layers

Description	Value
Start angle	-90
Revolution angle	225

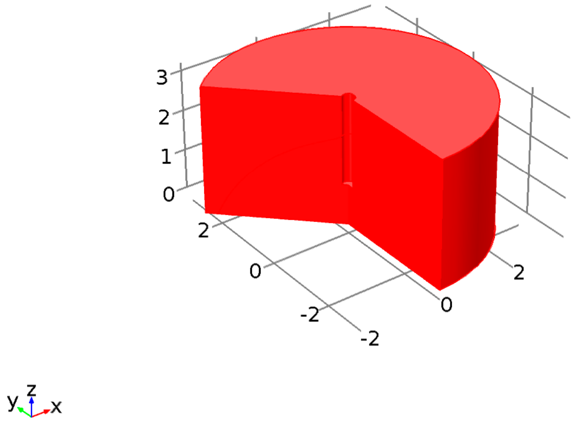

Data set: Revolution 2D 4

4.1.5 Study/Solution Store 1

Solution

Description	Value
Solution	Solution Store 1
Component	Geometry 1

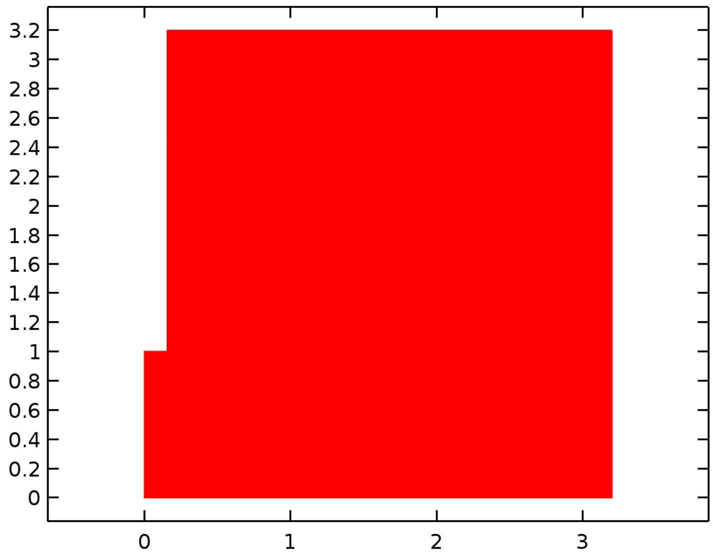

Data set: Study/Solution Store 1

4.1.6 Cut Point 2D 1

Data

Description	Value
Data set	Study/Solution 2

Point data

Description	Value
Entry method	Coordinates

Settings

Description	Value
r	2.5
z	0

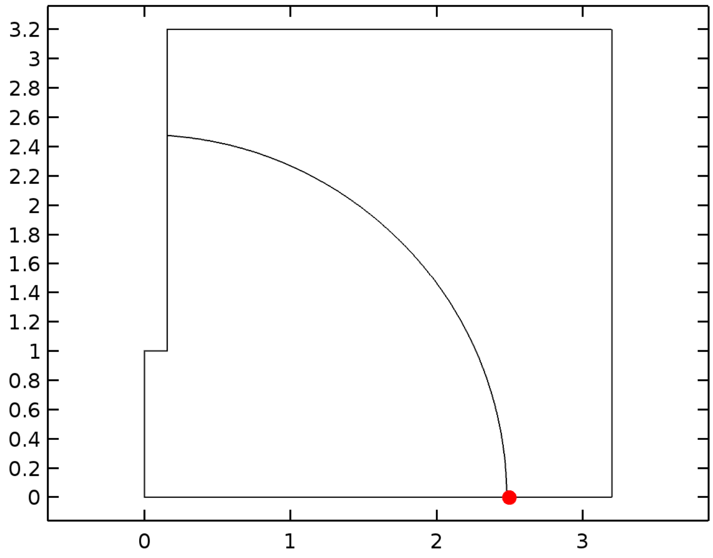

Data set: Cut Point 2D 1

4.2 Tables

4.2.1 Evaluation 2D

Interactive 2D values

Evaluation 2D

x	y	Value
0.72794	1.3388	0.0010187

4.3 Plot Groups

4.3.1 Velocity (tpfmm) 1

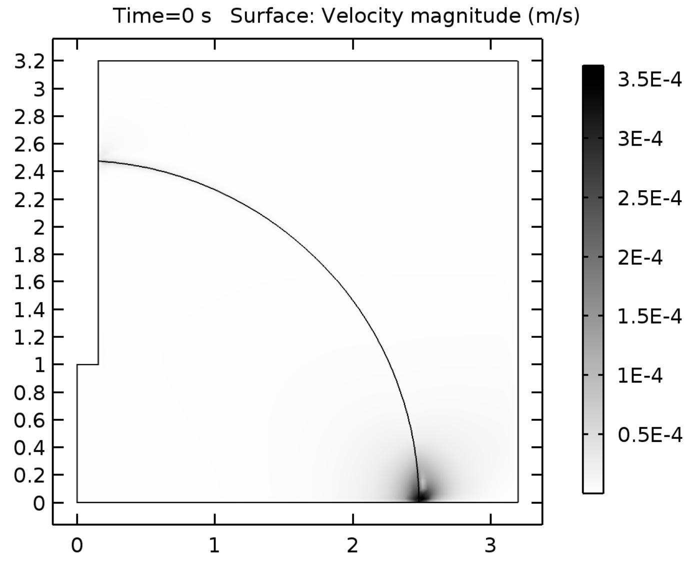

Time=0 s Surface: Velocity magnitude (m/s)

4.3.2 Pressure (tpfmm)

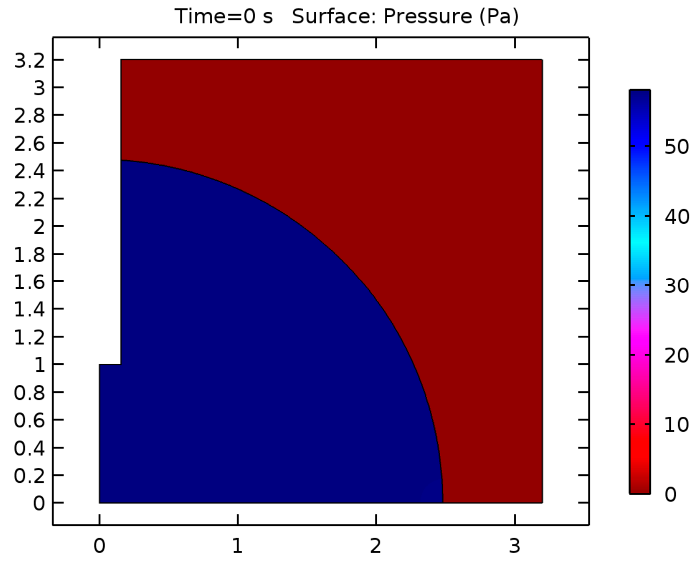

Time=0 s Surface: Pressure (Pa)

4.3.3 Density (tpfmm)

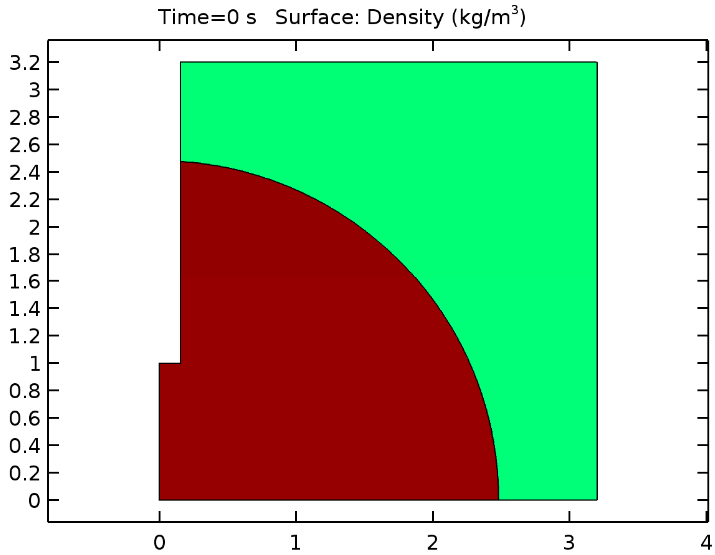

Time=0 s Surface: Density (kg/m³)

4.3.4 Velocity (tpfmm)

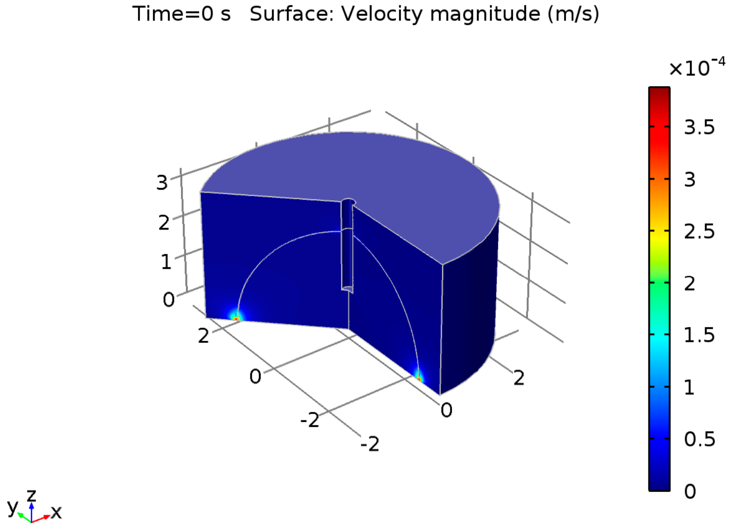

Time=0 s Surface: Velocity magnitude (m/s)

4.3.5 1D Plot Group 5

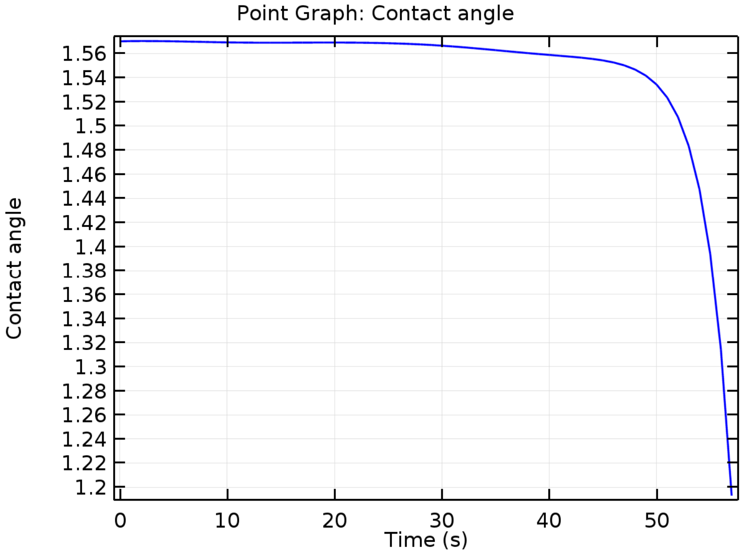

Point Graph: Contact angle
